# Terazosin Analogs Targeting Pgk1 as Neuroprotective Agents: Design, Synthesis, and Evaluation

**DOI:** 10.3389/fchem.2022.906974

**Published:** 2022-07-26

**Authors:** Yang Wang, Shihu Qian, Fang Zhao, Yujie Wang, Jiaming Li

**Affiliations:** ^1^ College of Pharmacy, Anhui University of Chinese Medicine, Hefei, China; ^2^ Institute of Medicinal Chemistry, Anhui Academy of Chinese Medicine, Hefei, China

**Keywords:** PGK1, agonist, nitrogen-containing heterocyclic compounds, neuroprotection, drug design

## Abstract

Nitrogen-containing heterocyclic compounds have shown promising therapeutic effects in a variety of inflammatory and neurodegenerative diseases. Recently, terazosin (TZ), a heterocyclic compound with a quinazoline core, was found to combine with phosphoglycerol kinase 1 (Pgk1) and protect neurons by enhancing Pgk1 activity and promoting glycolysis, thereby slowing, or preventing the neurodegeneration of PD. These findings indicated that terazosin analogs have bright prospects for the development of PD therapeutics. In this study, a series of terazosin analogs were designed and synthesized for neuroprotective effects by targeting Pgk1. Among them, compound **12b** was obtained with the best Pgk1 agonistic activity and neuroprotective activity. Further study indicates that it can increase intracellular ATP content and reduce ROS levels by stimulating the activity of Pgk1, thereby playing a role in protecting nerve cells. In conclusion, this study provides a new strategy and reference for the development of neuroprotective drugs.

## 1 Introduction

**FIGURE 1:**
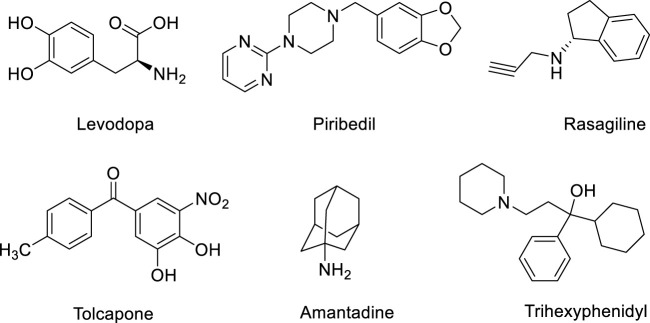
The main therapeutic anti-PD drugs.

Parkinson’s disease is a degenerative disease of the central nervous system characterized by degeneration and loss of dopaminergic neurons in the substantia nigra (Kalia and Lang 2015). At present, there is no effective treatment for the disease, and symptomatic treatment is mainly used in clinical practice (Liu et al., 2019; Raza et al., 2019). The main therapeutic drugs include dopamine substitutes (such as levodopa), dopamine receptor agonists (such as piribedil), monoamine oxidase-B (MAO-B) inhibitors (such as rasagiline), catechol-*O*-Methyltransferase (COMT) inhibitors (such as tolcapone), dopamine release promoting drugs (such as amantadine) and auxiliary drugs (such as trihexyphenidyl), etc. (Figure 1) (Cabreira and Massano 2019; Chakraborty et al., 2020). Although these therapies can alleviate the symptoms of PD to a certain extent, there is no drug that can reverse the neurodegenerative process of PD or prevent neurodegeneration (Carrera and Cacabelos 2019). Therefore, there is an urgent need to develop new PD therapeutic drugs with neuroprotective effects.

The pathogenesis of PD is not yet fully understood. Many studies supported the oxidative stress induced by reactive oxygen species (ROS), and the ATP generation disorder caused by mitochondrial dysfunction is closely related to the occurrence and development of PD (Yang et al., 2006; Jin et al., 2014; Szelechowski et al., 2014; Sironi et al., 2020). Various nitrogen-containing heterocyclic compounds can intervene in these processes and show significant effects in combating neuroinflammation and neurodegenerative diseases (Haghighijoo et al., 2022). Recently, using enzymology and X-ray crystallography, Liu Lei *et al.* found that terazosin (TZ) can combine with phosphoglycerol kinase 1 (Pgk1) to reduce organ damage and improve the survival rate of stroke in animal models (Chen et al., 2015). In further research, they found that terazosin can protect neurons by enhancing Pgk1 activity and promoting glycolysis, thereby slowing, or preventing PD neurodegeneration. Other related studies also showed that in animal models of PD such as mice, rats, and fruit flies, terazosin exhibited neuroprotective effects at low concentrations, and could slow or prevent neuronal dysfunction by increasing the level of ATP in the brain (Cai et al., 2019). These findings indicated that Pgk1 is a target for neuroprotection, and terazosin is a feasible lead for the development of neuroprotective drugs. At present, the research on terazosin analogs mainly focuses on α1 adrenergic receptor inhibitors (Desiniotis and Kyprianou 2011), and the research on small molecules with agonistic effects on Pgk1 is insufficient. The structure-activity relationship (SAR) between terazosin analogs and Pgk1 kinase is unclear and the reported compounds are less active (Chen et al., 2015). Therefore, we attempted to investigate the neuroprotective effects of terazosin analogs and discuss their SAR by introducing groups with different properties. These works will reveal whether terazosin analogs can exert neuroprotective effects by stimulating Pgk1.

## 2 Materials and Methods

### 2.1 Chemistry

The reagents and solvents for reaction were purchased from common commercial suppliers. If necessary, purification processes were carried out prior to their use. Melting points are determined on the melting point apparatus (RDCSY-I) and are uncorrected. ^1^H NMR and ^13^C NMR spectra were recorded on 400 and 100 MHz instruments (Bruker, Fallanden, Switzerland), respectively, with tetramethylsilane (TMS) as the internal standard. MS spectra were measured with a Hewlett-Packard 1100 LC/MSD spectrometer (Agilent, Waldbronn, Germany).

### 2.2 General Procedure for the Synthesis of Compounds 5a-n and 7a-f

Anhydrous piperazine (1.55g, 18 mmol, 6 eq) was dissolved in dichloromethane (10 ml). Benzyl chloride (0.38 g, 3 mmol, 1 eq) was added dropwise to the solution at room temperature. The reaction mixture was stirred for about 4 h. Then, the mixture was washed with saturated NaHCO_3_ solution (30 ml × 3) and water (30 ml × 3). The organic layer was dried over anhydrous Na_2_SO_4_. The filtrate was evaporated under reduced pressure and desired intermediate **3a**, yield 77.5%. Intermediates **3b-n** were synthesized according to the synthetic procedure given above.

Compound **3a** (0.175 g, 1.2 mmol, 1.0 eq) and compound **4** (0.24 g, 1.0 mmol, 1.0 eq) were dissolved in 1-pentanol (6 ml), and then heated under nitrogen protection Reflux at 135°C for 5 h. After the reaction, 1-pentanol was removed by evaporation under reduced pressure. The residue was purified by flash column chromatography afforded the compound **5a** 0.285 g, yield 75.1%. Compounds **5b-n** and **7a-f** were synthesized according to the synthetic procedure given above.

4-amino-6,7-dimethoxy-2-(4-benzylpiperazin-1-yl) quinazoline (5a): white solid, yield 75.1%, m. p. 145.1–147.4°C; ^1^H NMR (400 MHz, DMSO-*d*_*6*_) *δ* 7.43 (s, 1H, ArH), 7.38–7.24 (m, 5H, Ar**H**), 7.09 (s, 2H, ArN**H**_2_), 6.74 (s, 1H, Ar**H**), 3.83 (s, 3H, ArOC**H**_3_), 3.80 (s, 3H, ArOC**H**_3_), 3.76–3.70 (m, 4H, piperazine-**H**), 3.50 (s, 2H, ArC**H**_2_), 2.44–2.38 (m, 4H, piperazine-**H**). ^13^C NMR (101 MHz, DMSO-*d*_*6*_) *δ* 161.54, 158.94, 154.66, 149.28, 145.35, 138.67, 129.36, 128.66, 127.41, 105.64, 104.19, 103.32, 62.79, 56.31, 55.86, 53.26, 44.07. ESI-HRMS: m/z calcd for C_21_H_25_N_5_O_2_ [M + H]^+^ 380.2087, found 380.2098.

4-amino-6,7-dimethoxy-2-(4-(4-fluorobenzyl)piperazin-1-yl)quinazoline (5b): white solid, yield 52.6%, m. p. 194.9–196.0°C; ^1^H NMR (400 MHz, DMSO-*d*_*6*_) *δ* 7.43 (s, 1H, Ar**H**), 7.40–7.33 (m, 2H, ArH), 7.19–7.05 (m, 4H, ArH, ArNH2), 7.11 (s, 2H, ArN**H**_2_), 6.74 (s, 1H, Ar**H**), 3.83 (s, 3H, ArOC**H**_3_), 3.79 (s, 3H, ArOC**H**_3_), 3.75–3.69 (m, 4H, piperazine-**H**), 3.48 (s, 2H, ArC**H**_2_), 2.43–2.37 (m, 4H, piperazine-**H**). ^13^C NMR (101 MHz, DMSO-*d*_*6*_) *δ* 161.72 (*J*_*C-F*_ = 249.43 Hz), 161.54, 158.83, 154.67, 149.11, 145.37, 134.79 (*J*_*C-F*_ = 2.01 Hz), 131.17 (*J*_*C-F*_ = 8.04 Hz), 115.38 (*J*_*C-F*_ = 21.12 Hz), 105.55, 104.20, 103.31, 61.81, 56.30, 55.86, 53.12, 44.06. ESI-HRMS: m/z calcd for C_21_H_24_FN_5_O_2_ [M + H]^+^ 398.1992, found 398.1993.

4-amino-6,7-dimethoxy-2-(4-(4-chlorobenzyl)piperazin-1-yl)quinazoline (5c): white solid, yield 48.0%, m. p. 174.6–176.3°C; ^1^H NMR (400 MHz, DMSO-*d*_*6*_) *δ* 7.43 (s, 1H, Ar**H**), 7.41–7.34 (m, 4H, Ar**H**), 7.11 (s, 2H, ArN**H**_2_), 6.74 (s, 1H, Ar**H**), 3.83 (s, 3H, ArOC**H**_3_), 3.79 (s, 3H, ArOC**H**_3_), 3.75–3.69 (m, 4H, piperazine-**H**), 3.49 (s, 2H, ArC**H**_2_), 2.43–2.37 (m, 4H, piperazine-**H**). ^13^C NMR (101 MHz, DMSO-*d*_*6*_) *δ* 161.55, 158.82, 154.67, 149.11, 145.38, 137.77, 131.94, 131.10, 128.64, 105.56, 104.20, 103.31, 61.81, 56.31, 55.87, 53.17, 44.06. ESI-HRMS: m/z calcd for C_21_H_24_ClN_5_O [M + H]^+^ 414.1697, found 414.1694.

4-amino-6,7-dimethoxy-2-(4-(4-bromobenzyl)piperazin-1-yl)quinazoline (5d): white solid, yield 54.1%, m. p. 84.1–86.4°C; ^1^H NMR (400 MHz, DMSO-*d*_*6*_) *δ* 7.53 (d, *J* = 8.4 Hz, 2H, Ar**H**), 7.42 (s, 1H, Ar**H**), 7.31 (d, *J* = 8.4 Hz, 2H, Ar**H**), 7.12 (s, 2H, ArN**H**_2_), 6.73 (s, 1H, Ar**H**), 3.83 (s, 3H, ArOC**H**_3_), 3.79 (s, 3H, ArOC**H**_3_), 3.74–3.68 (m, 4H, piperazine-**H**), 3.48 (s, 2H, ArC**H**_2_), 2.43–2.38 (m, 4H, piperazine-**H**). ^13^C NMR (101 MHz, DMSO-*d*_*6*_) *δ* 161.55, 158.67, 154.69, 148.86, 145.41, 138.17, 131.57, 131.49, 120.44, 105.43, 104.22, 103.28, 61.83, 56.31, 55.88, 53.14, 44.06. ESI-HRMS: m/z calcd for C_21_H_24_BrN_5_O [M + H]^+^ 458.1192, found 458.1169.

4-amino-6,7-dimethoxy-2-(4-(4-methylbenzyl)piperazin-1-yl)quinazoline (5e): white solid, yield 38.1%, m. p. 173.5–175.1°C; ^1^H NMR (400 MHz, DMSO-*d*_*6*_) *δ* 7.42 (s, 1H, Ar**H**), 7.21 (d, *J* = 8.0 Hz, 2H, Ar**H**), 7.14 (d, *J* = 8.0 Hz, 2H, Ar**H**), 7.08 (s, 2H, ArN**H**_2_), 6.73 (s, 1H, Ar**H**), 3.83 (s, 3H, ArOC**H**_3_), 3.79 (s, 3H, ArOC**H**_3_), 3.74–3.68 (m, 4H, piperazine-**H**), 3.45 (s, 2H, ArC**H**_2_), 2.42–2.36 (m, 4H, piperazine-**H**), 2.29 (s, 3H, ArC**H**_3_). ^13^C NMR (101 MHz, DMSO-*d*_*6*_) *δ* 161.53, 158.93, 154.65, 149.29, 145.34, 136.41, 135.51, 129.35, 129.23, 105.63, 104.19, 103.31, 62.53, 56.30, 55.85, 53.19, 44.07, 21.17. ESI-HRMS: m/z calcd for C_22_H_27_N_5_O_2_ [M + H]^+^ 394.2243, found 394.2233.

4-amino-6,7-dimethoxy-2-(4-(4-methoxybenzyl)piperazin-1-yl)quinazoline (5f): white solid, yield 44.8%, m. p. 177.2–179.8°C; ^1^H NMR (400 MHz, DMSO-*d*_*6*_) *δ* 7.42 (s, 1H, Ar**H**), 7.24 (d, *J* = 8.4 Hz, 2H, Ar**H**), 7.08 (s, 2H, ArN**H**_2_), 6.90 (d, *J* = 8.4 Hz, 2H, Ar**H**), 6.73 (s, 1H, Ar**H**), 3.83 (s, 3H, ArOC**H**_3_), 3.79 (s, 3H, ArOC**H**_3_), 3.75 (s, 3H, ArOC**H**_3_), 3.73–3.68 (m, 4H, piperazine-**H**), 3.43 (s, 2H, ArC**H**_2_), 2.42–2.36 (m, 4H, piperazine-**H**). ^13^C NMR (101 MHz, DMSO-*d*_*6*_) *δ* 161.53, 158.92, 158.77, 154.66, 149.25, 145.34, 130.59, 130.40, 114.04, 105.62, 104.19, 103.31, 62.17, 56.30, 55.86, 55.46, 53.12, 44.06. ESI-HRMS: m/z calcd for C_22_H_27_N_5_O_3_ [M + H]^+^ 410.2192, found 410.2192.

4-amino-6,7-dimethoxy-2-(4-(4-(trifluoromethyl)piperazin-1-yl)quinazoline (5g): white solid, yield 55.9%, m. p. 93.7–95.4°C; ^1^H NMR (400 MHz, DMSO-*d*_*6*_) *δ* 7.70 (d, *J* = 7.6 Hz, 2H, Ar**H**), 7.58 (d, *J* = 7.6 Hz, 2H, Ar**H**), 7.43 (s, 1H, Ar**H**), 7.09 (s, 2H, ArN**H**_2_), 6.73 (s, 1H, Ar**H**), 3.83 (s, 3H, ArOC**H**_3_), 3.80 (s, 3H, ArOC**H**_3_), 3.76–3.71 (m, 4H, piperazine-**H**), 3.59 (s, 2H, ArC**H**_2_), 2.46–2.40 (m, 4H, piperazine-**H**). ^13^C NMR (101 MHz, DMSO-*d*_*6*_) *δ* 161.55, 158.92, 154.66, 149.30, 145.36, 143.86, 129.88, 128.11 (*J*_*C-F*_ = 32.18 Hz), 125.54 (*J*_*C-F*_ = 4.02 Hz), 124.85 (*J*_*C-F*_ = 271.56 Hz), 105.65, 104.18, 103.33, 62.02, 56.30, 55.85, 53.28, 44.05. ESI-HRMS: m/z calcd for C_22_H_24_F_3_N_5_O_2_ [M + H]^+^ 448.1960, found 448.1938.

4-amino-6,7-dimethoxy-2-(4-(4-cyanobenzyl)piperazin-1-yl)quinazoline (5h): white solid, yield 39.6%, m. p. 111.7–113.8°C; ^1^H NMR (400 MHz, DMSO-*d*_*6*_) *δ* 7.81 (d, *J* = 8.0 Hz, 2H, Ar**H**), 7.55 (d, *J* = 8.0 Hz, 2H, Ar**H**), 7.42 (s, 1H, Ar**H**), 7.09 (s, 2H, ArN**H**_2_), 6.73 (s, 1H, Ar**H**), 3.83 (s, 3H, ArOC**H**_3_), 3.79 (s, 3H, ArOC**H**_3_), 3.76–3.71 (m, 4H, piperazine-**H**), 3.59 (s, 2H, ArC**H**_2_), 2.45–2.39 (m, 4H, piperazine-**H**). ^13^C NMR (101 MHz, DMSO-*d*_*6*_) *δ* 161.55, 158.88, 154.66, 149.25, 145.37, 144.95, 132.65, 130.07, 119.39, 110.21, 105.62, 104.18, 103.33, 62.04, 56.30, 55.86, 53.25, 44.05. ESI-HRMS: m/z calcd for C_22_H_24_N_6_O_2_ [M + H]^+^ 405.2039, found 405.2017.

4-amino-6,7-dimethoxy-2-(4-(4-nitrobenzyl)piperazin-1-yl)quinazoline (5i): yellow solid, yield 56.5%, m. p. 97.5–99.2°C; ^1^H NMR (400 MHz, DMSO-*d*_*6*_) *δ* 8.21 (d, *J* = 8.4 Hz, 2H, Ar**H**), 7.63 (d, *J* = 8.4 Hz, 2H, Ar**H**), 7.42 (s, 1H, Ar**H**), 7.10 (s, 2H, ArN**H**_2_), 6.73 (s, 1H, Ar**H**), 3.83 (s, 3H, ArOC**H**_3_), 3.79 (s, 3H, ArOC**H**_3_), 3.76–3.70 (m, 4H, piperazine-**H**), 3.64 (s, 2H, ArC**H**_2_), 2.46–2.40 (m, 4H, piperazine-**H**). ^13^C NMR (101 MHz, DMSO-*d*_*6*_) *δ* 161.55, 158.81, 154.67, 149.13, 147.19, 147.07, 145.38, 130.22, 123.85, 105.57, 104.18, 103.31, 61.72, 56.30, 55.86, 53.26, 44.05. EESI-HRMS: m/z calcd for C_21_H_24_N_6_O_4_ [M + H]^+^ 425.1937, found 425.1926.

4-amino-6,7-dimethoxy-2-(4-(4-(methylsulfonyl)benzyl)piperazin-1-yl)quinazoline (5j): white solid, yield 52.3%, m. p. 115.2–116.5°C; ^1^H NMR (400 MHz, DMSO-*d*_*6*_) *δ* 7.91 (d, *J* = 8.4 Hz, 2H, Ar**H**), 7.62 (d, *J* = 8.4 Hz, 2H, Ar**H**), 7.42 (s, 1H, Ar**H**), 7.11 (s, 2H, ArN**H**_2_), 6.73 (s, 1H, Ar**H**), 3.83 (s, 3H, ArOC**H**_3_), 3.79 (s, 3H, ArOC**H**_3_), 3.76–3.71 (m, 4H, piperazine-**H**), 3.61 (s, 2H, ArC**H**_2_), 3.22 (s, 3H, ArSO_2_C**H**_3_), 2.46–2.41 (m, 4H, piperazine-**H**). ^13^C NMR (101 MHz, DMSO-*d*_*6*_) *δ* 161.55, 158.82, 154.67, 149.14, 145.38, 145.10, 139.94, 129.95, 127.43, 105.57, 104.18, 103.31, 61.97, 56.30, 55.87, 53.28, 44.06, 44.04. ESI-HRMS: m/z calcd for C_22_H_27_N_5_O_4_S [M + H]^+^ 458.1862, found 458.1852.

4-amino-6,7-dimethoxy-2-(4-(2-methoxybenzyl)piperazin-1-yl)quinazoline (5k): white solid, yield 64.6%, m. p. 84.8–86.4°C; ^1^H NMR (400 MHz, DMSO-*d*_*6*_) *δ* 7.42 (s, 1H, Ar**H**), 7.36 (dd, *J* = 7.6, 1.6 Hz, 1H, Ar**H**), 7.26–7.21 (m, 1H, Ar**H**), 7.07 (s, 2H, ArN**H**_2_), 6.98 (d, *J* = 8.4 Hz, 1H, Ar**H**), 6.96–6.92 (m, 1H, Ar**H**), 6.72 (s, 1H, Ar**H**), 3.83 (s, 3H, ArOC**H**_3_), 3.79 (s, 3H, ArOC**H**_3_), 3.78 (s, 3H, ArOC**H**_3_), 3.75–3.70 (m, 4H, piperazine-H), 3.51 (s, 2H, ArC**H**_2_), 2.46–2.41 (m, 4H, piperazine-**H**). ^13^C NMR (101 MHz, DMSO-*d*_*6*_) *δ* 161.53, 158.89, 157.87, 154.65, 149.32, 145.30, 130.33, 128.47, 126.17, 120.58, 111.26, 105.62, 104.19, 103.28, 56.30, 56.13, 55.85, 55.78, 53.40, 44.07. ESI-HRMS: m/z calcd for C_22_H_27_N_5_O_3_ [M + H]^+^ 410.2192, found 410.2189.

4-amino-6,7-dimethoxy-2-(4-(3-methoxybenzyl)piperazin-1-yl)quinazoline (5l): white solid, yield 65.3%, m. p. 63.8–65.4°C; ^1^H NMR (400 MHz, DMSO-*d*_*6*_) *δ* 7.42 (s, 1H, Ar**H**), 7.25 (t, *J* = 8.0 Hz, 1H, Ar**H**), 7.07 (s, 2H, ArN**H**_2_), 6.91 (d, *J* = 7.6 Hz, 2H, Ar**H**), 6.84–6.81 (m, 1H, Ar**H**), 6.73 (s, 1H, Ar**H**), 3.83 (s, 3H, ArOC**H**_3_), 3.79 (s, 3H, ArOC**H**_3_), 3.75 (s, 3H, ArOC**H**_3_), 3.74–3.69 (m, 4H, piperazine-**H**), 3.47 (s, 2H, ArC**H**_2_), 2.43–2.38 (m, 4H, piperazine-**H**). ^13^C NMR (101 MHz, DMSO-*d*_*6*_) *δ* 161.53, 159.73, 158.95, 154.65, 149.32, 145.33, 140.34, 129.68, 121.52, 114.81, 112.76, 105.64, 104.17, 103.31, 62.69, 56.30, 55.85, 55.40, 53.26, 44.07. ESI-HRMS: m/z calcd for C_22_H_27_N_5_O_3_ [M + H]^+^ 410.2192, found 410.2188.

4-amino-6,7-dimethoxy-2-(4-(2-chlorobenzyl)piperazin-1-yl)quinazoline (5m): white solid, yield 65.9%, m.p. 132.6–135.1°C; ^1^H NMR (400 MHz, DMSO-*d*_*6*_) *δ* 7.55 (dd, *J* = 7.2, 1.2 Hz, 1H, Ar**H**), 7.48 (s, 1H, Ar**H**), 7.44 (dd, *J* = 7.6, 1.2 Hz, 1H, Ar**H**), 7.41–7.26 (m, 4H, Ar**H**, ArN**H**_2_), 6.85 (s, 1H, Ar**H**), 3.84 (s, 3H, ArOC**H**_3_), 3.80 (s, 3H, ArOC**H**_3_), 3.78–3.73 (m, 4H, piperazine-**H**), 3.61 (s, 2H, ArC**H**_2_), 2.50–2.46 (m, 4H, piperazine-**H**). ^13^C NMR (101 MHz, DMSO-*d*_*6*_) *δ* 161.60, 157.69, 154.84, 145.67, 135.93, 133.84, 131.41, 130.10, 129.76, 129.13, 127.52, 104.62, 104.42, 103.12, 59.22, 56.38, 55.96, 53.19, 44.19. ESI-HRMS: m/z calcd for C_21_H_24_ClN_5_O [M + H]^+^ 414.1697, found 414.1688.

4-amino-6,7-dimethoxy-2-(4-(3-chlorobenzyl)piperazin-1-yl)quinazoline (5n): white solid, yield 62.1%, m. p. 119.9–121.0°C; ^1^H NMR (400 MHz, DMSO-*d*_*6*_) *δ* 7.52 (s, 2H, Ar**H**), 7.43–7.30 (m, 5H, Ar**H**, ArN**H**_2_), 6.94 (s, 1H, Ar**H**), 3.84 (s, 3H, ArOC**H**_3_), 3.80 (s, 3H, ArOC**H**_3_), 3.79–3.73 (m, 4H, piperazine-**H**), 3.56 (s, 2H, ArC**H**_2_), 2.49–2.44 (m, 4H, piperazine-**H**). ^13^C NMR (101 MHz, DMSO-*d*_*6*_) *δ* 161.62, 156.94, 154.94, 145.89, 141.02, 133.46, 130.58, 130.11, 129.07, 128.07, 127.56, 104.55, 104.01, 103.02, 61.58, 56.43, 56.03, 52.83, 44.17. ESI-HRMS: m/z calcd for C_21_H_24_ClN_5_O [M + H]^+^ 414.1697, found 414.1689.

4-amino-6,7-dimethoxy-2-(4-(4-benzoyl)piperazin-1-yl)quinazoline (7a): white solid, yield 68.7%, m. p. 268.0–269.5°C; ^1^H NMR (400 MHz, DMSO-*d*_*6*_) *δ* 7.49–7.43 (m, 6H, Ar**H**), 7.17 (s, 2H, ArN**H**_2_), 6.75 (s, 1H, Ar**H**), 3.83 (s, 3H, ArOC**H**_3_), 3.80 (s, 3H, ArOC**H**_3_), 3.76–3.37 (m, 8H, piperazine-**H**). ^13^C NMR (101 MHz, DMSO-*d*_*6*_) *δ* 169.64, 161.63, 158.70, 154.73, 149.05, 145.56, 136.47, 130.01, 128.90, 127.50, 105.62, 104.17, 103.46, 56.31, 55.89, 55.37, 44.14. ESI-HRMS: m/z calcd for C_21_H_23_N_5_O_3_ [M + H]^+^ 394.1879, found 394.1872.

4-amino-6,7-dimethoxy-2-(4-((2,1,3-benzoxadiazole-5-yl)methyl)piperazin-1-yl)quinazoline (7b): yellow solid, yield 42.7%, m. p. 177.9–179.5°C; ^1^H NMR (400 MHz, DMSO-*d*_*6*_) *δ* 8.02 (d, *J* = 9.2 Hz, 1H, Ar**H**), 7.90 (s, 1H, Ar**H**), 7.63 (d, *J* = 9.2 Hz, 1H, Ar**H**), 7.43 (s, 1H, Ar**H**), 7.11 (s, 2H, ArN**H**_2_), 6.74 (s, 1H, Ar**H**), 3.84 (s, 3H, ArOC**H**_3_), 3.80 (s, 3H, ArOC**H**_3_), 3.78–3.70 (m, 4H, piperazine-**H**), 3.62 (s, 2H, ArC**H**_2_), 2.50–2.43 (m, 4H, piperazine-**H**). ^13^C NMR (101 MHz, DMSO-*d*_*6*_) *δ* 161.55, 158.83, 154.66, 149.52, 149.16, 145.37, 144.32, 134.99, 116.25, 114.03, 105.59, 104.18, 103.32, 62.05, 56.30, 55.86, 53.29, 44.06. ESI-HRMS: m/z calcd for C_21_H_23_N_7_O_3_ [M + H]^+^ 422.1941, found 422.1936.

4-amino-6,7-dimethoxy-2-(4-(1,3-benzodioxol-5-ylmethyl)piperazin-1-yl)quinazoline (7c): white solid, yield 47.2%, m. p. 96.2–97.6°C; ^1^H NMR (400 MHz, DMSO-*d*_*6*_) *δ* 7.42 (s, 1H, Ar**H**), 7.08 (s, 2H, ArN**H**_2_), 6.90 (s, 1H, Ar**H**), 6.86 (d, *J* = 8.0 Hz, 1H, Ar**H**), 6.78 (d, *J* = 8.0 Hz, 1H, Ar**H**), 6.73 (s, 1H, Ar**H**), 6.00 (s, 2H, OC**H**_2_O), 3.83 (s, 3H, ArOC**H**_3_), 3.79 (s, 3H, ArOC**H**_3_), 3.74–3.66 (m, 4H, piperazine-**H**), 3.41 (s, 2H, ArC**H**_2_), 2.43–2.32 (m, 4H, piperazine-**H**). ^13^C NMR (101 MHz, DMSO-*d*_*6*_) *δ* 161.53, 158.92, 154.65, 149.28, 147.70, 146.64, 145.33, 132.48, 122.47, 109.58, 108.32, 105.63, 104.19, 103.31, 101.24, 62.44, 56.30, 55.85, 53.09, 44.07. ESI-HRMS: m/z calcd for C_22_H_25_N_5_O_4_ [M + H]^+^ 424.1985, found 424.1983.

4-amino-6,7-dimethoxy-2-(4-(2-hydroxyethyl)piperazin-1-yl)quinazoline (7d): white solid, yield 42.0%, m. p. 50.2–52.8°C; ^1^H NMR (400 MHz, DMSO-*d*_*6*_) *δ* 7.43 (s, 1H, Ar**H**), 7.11 (s, 2H, ArN**H**_2_), 6.74 (s, 1H, Ar**H**), 4.48 (s, 1H, O**H**), 3.83 (s, 3H, ArOC**H**_3_), 3.79 (s, 3H, ArOC**H**_3_), 3.74–3.68 (m, 4H, piperazine-**H**), 3.56 (t, *J* = 6.1 Hz, 2H, CH_2_C**H**_2_OH), 2.51 (t, *J* = 6.1 Hz, 2H, C**H**_2_CH_2_OH), 2.49–2.42 (m, 4H, piperazine-**H**). ^13^C NMR (101 MHz, DMSO-*d*_*6*_) *δ* 161.54, 158.84, 154.66, 149.09, 145.37, 105.55, 104.19, 103.31, 60.80, 58.77, 56.31, 55.86, 53.66, 43.92. ESI-HRMS: m/z calcd for C_16_H_23_N_5_O_3_ [M + H]^+^ 334.1879, found 334.1872.

4-amino-6,7-dimethoxy-2-(4-(2-methoxyethyl)piperazin-1-yl)quinazoline (7e): white solid, yield 57.6%, m. p. 77.8–79.1°C; ^1^H NMR (400 MHz, DMSO-*d*_*6*_) *δ* 7.41 (s, 1H, Ar**H**), 7.07 (s, 2H, ArN**H**_2_), 6.73 (s, 1H, Ar**H**), 3.83 (s, 3H, ArOC**H**_3_), 3.79 (s, 3H, ArOC**H**_3_), 3.71–3.66 (m, 4H, piperazine-**H**), 3.47 (t, *J* = 5.9 Hz, 2H, CH_2_C**H**_2_OCH_3_), 3.25 (s, 3H, CH_2_CH_2_OC**H**_3_), 2.50 (t, *J* = 5.6 Hz, 2H, C**H**_2_CH_2_OCH_3_), 2.46–2.42 (m, 4H, piperazine-**H**). ^13^C NMR (101 MHz, DMSO-*d*_*6*_) *δ* 161.52, 158.96, 154.64, 149.28, 145.33, 105.63, 104.17, 103.30, 70.34, 58.47, 57.73, 56.30, 55.85, 53.74, 44.07. ESI-HRMS: m/z calcd for C_17_H_25_N_5_O_3_ [M + H]^+^ 348.2036, found 348.2025.

4-amino-6,7-dimethoxy-2-(4-(acetamido-2-yl)piperazin-1-yl)quinazoline (7f): white solid, yield 43.3%, m. p. 146.7–148.4°C; ^1^H NMR (400 MHz, DMSO-*d*_*6*_) *δ* 7.42 (s, 1H, Ar**H**), 7.26–7.10 (m, 4H, ArN**H**_2_), 6.74 (s, 1H, Ar**H**), 3.83 (s, 3H, ArOC**H**_3_), 3.79 (s, 3H, ArOC**H**_3_), 3.77–3.72 (m, 4H, piperazine-H), 2.90 (s, 2H, COCH2), 2.49–2.45 (m, 4H, piperazine-H). ^13^C NMR (101 MHz, DMSO-*d*_*6*_) *δ* 172.07, 161.55, 158.69, 154.68, 148.91, 145.40, 105.44, 104.20, 103.26, 61.86, 56.31, 55.88, 53.44, 43.96. ESI-HRMS: m/z calcd for C_16_H_22_N_6_O_3_ [M + H]^+^ 347.1832, found 347.1813.

### 2.3 General Procedure for the Synthesis of Compounds 10a-c

Compound **8** (0.518 g, 2.0 mmol, 1.0 eq) was dissolved in ethanol (6 ml) and dichloromethane (6 ml). Then sodium borohydride (0.114 g, 3.0 mmol, 1.5 eq) was added to the reaction solution in batches, and the reaction was carried out at room temperature for 24 h. After the reaction, ethanol and dichloromethane were removed by evaporation under reduced pressure to obtain the crude compound **9**. The crude compound **9** was dissolved in dichloromethane (100 ml) and washed with saturated NaCl solution (50 ml × 3). The organic layer was dried over anhydrous Na_2_SO_4_ and evaporated under reduced pressure. The residue was purified by flash column chromatography afforded the compound **9** 0.2 g, yield 44.5%.

Compound **9** (0.112 g, 0.5 mmol, 1 eq) and N-(2-tetrahydrofuroyl)piperazine (0.08 ml, 0.5 mmol, 1 eq) were dissolved in 1-pentanol (5 ml), and then heated under nitrogen protection Reflux at 135°C for 5 h. After the reaction, 1-pentanol was removed by evaporation under reduced pressure. The residue was purified by flash column chromatography afforded the compound **10a** 0.12 g, yield 64.4%. Compounds **10b-c** were synthesized according to the synthetic procedure given above.

6,7-dimethoxy-2-(4-(tetrahydrofuran-2-carbonyl)piperazin-1-yl)quinazoline (10a): yellow solid, yield 64.4%, m. p. 128.5–130.8°C; ^1^H NMR (400 MHz, DMSO-*d*_*6*_) *δ* 8.97 (s, 1H, Ar**H**), 7.25 (s, 1H, Ar**H**), 6.95 (s, 1H, Ar**H**), 4.75–4.71 (m, 1H, COCH), 3.92 (s, 3H, ArOC**H**_3_), 3.85 (s, 3H, ArOC**H**_3_), 3.84–3.71 (m, 6H, CH_2_CH_2_C**H**_2_O, piperazine-**H**), 3.67–3.54 (m, 4H, piperazine-**H**), 2.11–2.00 (m, 2H, CH_2_C**H**_2_CH_2_O), 1.90–1.81 (m, 2H, C**H**_2_CH_2_CH_2_O). ^13^C NMR (101 MHz, DMSO-*d*_*6*_) *δ* 170.03, 159.05, 158.76, 156.71, 149.54, 147.24, 114.75, 105.90, 104.93, 75.42, 68.70, 56.33, 56.05, 45.08, 44.51, 44.01, 41.76, 28.53, 25.73. ESI-HRMS: m/z calcd for C_19_H_24_N_4_O_4_ [M + H]^+^ 373.1876, found 373.1860.

6,7-dimethoxy-2-(4-benzylpiperazin-1-yl)quinazoline (10b): yellow solid, yield 61.5%, m. p. 91.2–92.8°C; ^1^H NMR (400 MHz, DMSO-*d*_*6*_) *δ* 8.93 (s, 1H, Ar**H**), 7.37–7.33 (m, 4H, ArH), 7.31–7.25 (m, 4.2 Hz, 1H, ArH), 7.22 (s, 1H, Ar**H**), 6.91 (s, 1H, Ar**H**), 3.90 (s, 3H, ArOC**H**_3_), 3.84 (s, 3H, ArOC**H**_3_), 3.83–3.73 (m, 4H, piperazine-**H**), 3.52 (s, 2H, ArC**H**_2_), 2.49–2.40 (m, 4H, piperazine-**H**). ^13^C NMR (101 MHz, DMSO-*d*_*6*_) *δ* 158.95, 158.93, 156.61, 149.63, 147.08, 129.42, 129.33, 128.70, 127.51, 114.59, 105.90, 104.93, 62.58, 56.28, 56.03, 52.98, 44.14. ESI-HRMS: m/z calcd for C_21_H_24_N_4_O_2_ [M + H]^+^ 365.1978, found 365.1968.

6,7-dimethoxy-2-(4-((2,1,3-benzoxadiazole-5-yl)methyl)piperazin-1-yl)quinazoline (10c): yellow solid, yield 55.2%, m. p. 160.1–162.8°C; ^1^H NMR (400 MHz, DMSO-*d*_*6*_) *δ* 8.94 (s, 1H, Ar**H**), 8.03 (d, *J* = 9.3 Hz, 1H, Ar**H**), 7.93 (s, 1H, Ar**H**), 7.65 (d, *J* = 9.3 Hz, 1H, Ar**H**), 7.22 (s, 1H, Ar**H**), 6.92 (s, 1H, Ar**H**), 3.90 (s, 3H, ArOC**H**_3_), 3.88–3.78 (m, 7H, ArOC**H**_3_, piperazine-**H**), 3.67 (s, 2H, ArC**H**_2_), 2.52–2.47 (m, 4H, piperazine-**H**). ^13^C NMR (101 MHz, DMSO-*d*_*6*_) *δ* 158.99, 158.93, 156.63, 149.63, 149.53, 149.19, 147.11, 144.25, 135.00, 116.31, 114.60, 114.10, 105.92, 104.93, 61.93, 56.32, 56.05, 53.10, 44.22. ESI-HRMS: m/z calcd for C_21_H_22_N_6_O_3_ [M + H]^+^ 407.1832, found 407.1815.

### 2.4 General Procedure for the Synthesis of Compounds 12a and 12b

Compound **11** (0.115 g, 0.7 mmol, 1 eq) was dissolved in dichloromethane (5 ml), then K_2_CO_3_ (0.1 g, 1.4 mmol, 2 eq), 10 mg KI and 2-(chloromethyl)-3,5,6-trimethylpyrazine (0.12 g, 0.7 mmol, 1 eq) were added. The mixture was reacted at room temperature for 8 h. Dichloromethane (50 ml) was added to the reaction solution, washed with saturated sodium chloride solution (30 ml × 3). The organic layer was dried over anhydrous Na_2_SO_4_ and evaporated under reduced pressure. The residue was purified by flash column chromatography afforded the compound **12a** 0.18 g, yield 86.5%. Compound **12b** was synthesized according to the synthetic procedure given above.

2-(4-((3,5,6-trimethylpyrazin-2-yl)methyl)piperazin-1-yl)pyrimidine (12a): white solid, yield 86.5%, m.p. 57.8–60.1°C; ^1^H NMR (400 MHz, DMSO-*d*_*6*_) *δ* 8.34 (d, *J* = 4.7 Hz, 2H, Ar**H**), 6.60 (t, *J* = 4.7 Hz, 1H, Ar**H**), 3.70–3.65 (m, 4H, piperazine-**H**), 3.58 (s, 2H, ArC**H**_2_), 2.54–2.49 (m, 4H, piperazine-**H**), 2.45–2.40 (m, 9H, C**H**_3_). ^13^C NMR (101 MHz, DMSO-*d*_*6*_) *δ* 161.64, 158.32, 150.02, 149.83, 147.92, 147.80, 110.53, 61.95, 53.06, 43.77, 21.54, 21.45, 21.00. ESI-HRMS: m/z calcd for C_16_H_22_N_6_ [M + H]^+^ 299.1984, found 299.1981.

2- (4-((2,1,3-benzoxadiazole-5-yl)methyl)piperazin-1-yl)pyrimidine (12b): white solid, yield 66.3%, m. p. 115.5–117.9°C; ^1^H NMR (400 MHz, DMSO-*d*_*6*_) *δ* 8.36 (d, *J* = 4.7 Hz, 2H, Ar**H**), 8.02 (d, *J* = 9.2 Hz, 1H, Ar**H**), 7.91 (s, 1H, Ar**H**), 7.64 (d, *J* = 9.3 Hz, 1H, Ar**H**), 6.63 (t, *J* = 4.6 Hz, 1H, Ar**H**), 3.79–3.74 (m, 4H, piperazine-**H**), 3.65 (s, 2H, ArC**H**_2_), 2.53–2.48 (m, 4H, piperazine-**H**). ^13^C NMR (101 MHz, DMSO-*d*_*6*_) *δ* 161.66, 158.35, 149.51, 149.15, 144.15, 134.95, 116.30, 114.10, 110.59, 61.84, 52.92, 43.77. ESI-HRMS: m/z calcd for C_15_H_16_N_6_O [M + H]^+^ 297.1464, found 297.1492.

### 2.5 Docking Study

This part was completed using Discovery Studio 2017R2 with the help of Professor Liu Xinhua from Anhui Medical University. The co-crystal structure of Pgk1 and terazosin was obtained from the PDB database (PDB code: 4O3F). The docking site was defined by the ligand in the co-crystal, and the CDOCKER module was used to conduct the molecular docking study of small molecules and Pgk1.

### 2.6 Cell Culture

SH-SY5Y cell line was purchased from American Tissue Culture Collection (ATCC, Rockville, MD, USA). Terazosin was purchased from GlpBio Technology (Montclair, CA, USA). Piribedil was purchased from MedChemExpress (MCE, Shanghai, China). Recombinant human Pgk1 protein (rhPgk1), MPP^+^ were purchased from Abcam (Cambridge, United Kingdom). DL-Glyceraldehyde 3-Phosphate (GAP), DTT, Glyceraldehyde 3-Phosphate Dehydrogenase (GAPDH), beta-Nicotinamide adenine dinucleotide (β-NAD) and ADP were purchased from Sigma-Aldrich (Darmstadt, Germany).

### 2.7 Cell Viability Assay

SH-SY5Y cells were cultured in 96-well plates, 3,000 cells per well. The next day, the medium was changed to a serum-free DMEM medium containing the corresponding concentration of the test compounds. After 3 h of incubation, MPP^+^ was added at a final concentration of 2 mM. The plates were incubated for an additional 48 h, and 20 μL of MTT (5 mg/ml) was added to each well 4 h before the end of the incubation. Finally, the culture medium was removed and 150 μL of DMSO was added to each well. Shaken the plates on a cell shaker for 10 min to completely dissolve the crystals, and the OD_570_ was measured with a microplate reader. Mean values and standard deviations were from 3 independent experiments. Cell survival rate (%) = (OD _sample_- OD _blank_)/(OD _control_ - OD _blank_) × 100%.

### 2.8 Pgk1 Agonistic Activity Assay

The positive response of Pgk1 can lead to the reduction of ADP levels, promoting the production of NADH, which can be detected at the absorbance wavelength of 340 nm. Thus, the change in NADH levels from baseline (ΔOD) can be indicative of Pgk1 activity. Reaction buffer contains 20 mM Tris-HCl pH7.6, 100 mM NaCl, 5 mM MgCl_2_, 2 mM DTT. Before the reaction, 1 mM GAP, 0.3 mM β-NAD, 4 U GAPDH and 0.02 ng/μL of rhPgk1 protein were added. Mix the above reaction mixture evenly, and add 75 μL of the above mixture to a 96-well assay plate, 75 μL per well. Afterward, 20 μL of 250 nM test compound (prepared with reaction buffer, the final concentration at 50 nM) was added to each well, and the control was added with the same amount of solvent. The reaction was started by adding 5 μL of 4 mM ADP (final concentration at 0.2 mM) per well after 10 min incubation at room temperature. OD_340_ was measured at 0 and 30 min of the reaction, respectively. Mean values and standard deviations were from 3 independent experiments. Relative Pgk1 activity (%) = (ΔOD _sample_/ΔOD _control_) ×100%.

### 2.9 Determination of ATP Level in SH-SY5Y Cells

CellTiter-Glo cell viability detection kit was used to detect the ATP content in the cells. The preparation of cells is similar to Section 2.4. After 48 h of incubation, an equal volume of freshly prepared CellTiter-Glo reagent was added to each well for 30 min. Mixed the contents on an orbital shaker for 2 minutes to fully lyse the cells. After the plate was incubated at room temperature for 10 min, the luminescence signal was detected, and the ATP concentration of each group was calculated. Relative ATP level (%) = (Lum _sample_- Lum _blank_)/(Lum _control_ - Lum _blank_) × 100%.

### 2.10 Determination of ROS Level in SH-SY5Y Cells

SH-SY5Y cells in the logarithmic growth phase were selected and plated in a 6-well plate at a concentration of 2 × 10^5^ cells/well. At the same time, the control group, MPP^+^ model group, terazosin group and target compound group were set up. After the cells adhered, the original medium was discarded, the terazosin group and the target compound group were added with corresponding volumes of serum-free medium containing the test compound, and the control group and the MPP^+^ model group were added with the same amount of serum-free medium. After incubating at 37°C, 5% CO_2_ for 1 h, MPP^+^ was added to make the final concentration 2 mM, and the control group was added with a corresponding volume of PBS. After cultivating for 48 h, Biyuntian’s reactive oxygen detection kit (S0033S) was used to detect the content of reactive oxygen species.

## 3 Results and Discussion

### 3.1 Design and Synthesis

**FIGURE 2:**
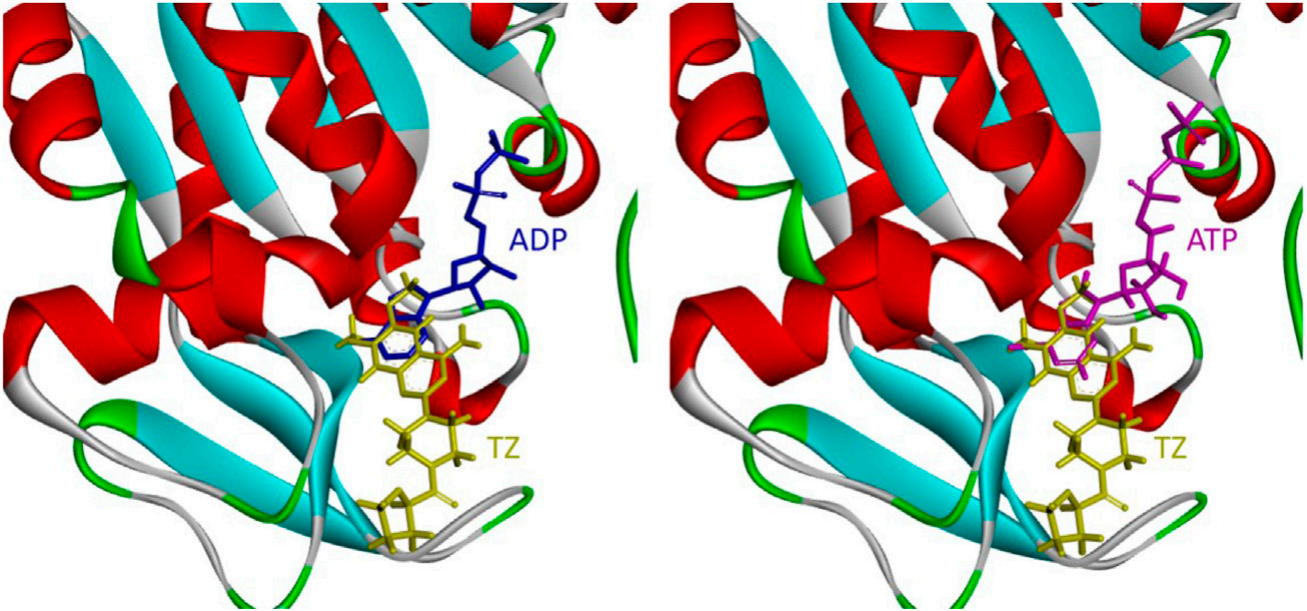
Relative positions of TZ, ADP or ATP in the active pocket of Pgk1 (PDB ID: 2X15).

During glucose metabolism, ADP enters the cleft of the active pocket of Pgk1 and is converted into ATP. In this process, terazosin can promote the release of ATP by competing for the binding site, re-exposing the binding pocket, thereby exerting an agonistic effect (Figure 2) (Chen et al., 2015). This requires the small molecule to have an appropriate affinity for Pgk1 and avoid strong interactions with residues around the ADP binding site in the cleft, otherwise, ADP will not be able to enter the active pocket and inhibit the activity of Pgk1 (Jo et al., 2021). Analyzing the co-crystal structure of TZ and Pgk1, we found that there are three hydrophobic rings (quinazoline ring, piperazine ring and tetrahydrofuran ring) in the structure of terazosin surrounded by multiple hydrophobic residues of Pgk1. Among them, the phenyl ring of the quinazoline core occupies the same site as the pyrimidine ring of the purine in ADP or ATP, and both are inserted into the same hydrophobic pocket in the C-terminal domain of the Pgk1 (Chen et al., 2015; Xia et al., 2017). Therefore, overly robust residue interactions should not be added to this region to avoid compounds occupying the ADP binding site and inhibiting the function of Pgk1. The pyrimidine ring forms a π-π stacking interaction with the Phe292 residue, which is a relatively strong interaction. The piperazine ring and tetrahydrofuran carboxamide group are located at the entrance to the active pocket, surrounded by the Leu257, Phe292, Met312 and Leu314 amino acid residues of Pgk1. Among them, the piperazine group forms a π-sigma interaction with Phe292, and the tetrahydrofuran carboxamide group has potential hydrogen bond interactions with the Phe292 or Thr255 residues of Pgk1, which maintain the binding of terazosin and Pgk1. Compared with the methoxy groups at the C-6 and C-7 positions of the terazosin-quinazoline ring, the C-2 position has a greater degree of freedom in structural modification and is an ideal modification site. In addition, the amino group at the C-4 position lacks interaction with the receptor and is also a potential modification site. Therefore, we firstly modified the tetrahydrofuran carboxamide group at the C-2 position and the amino group at the C-4 position of terazosin in order to develop new Pgk1 agonists for neuroprotective agents.

**SCHEME 1:**
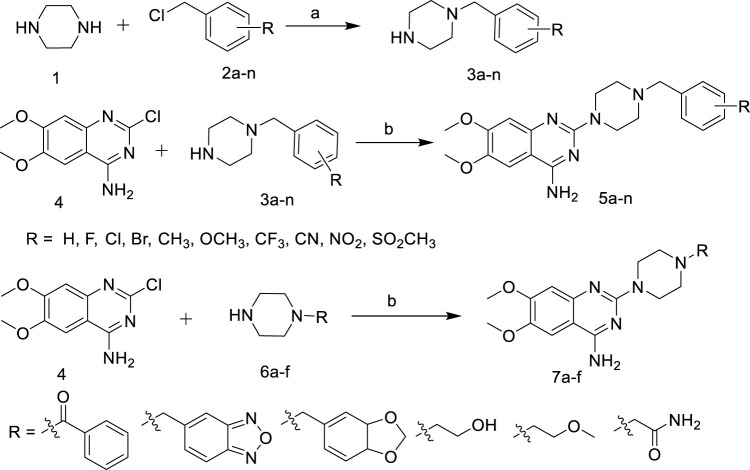
Synthetic of target compounds **5a ∼ n** and **7a ∼ f**. Reagents and conditions: (a) CH_2_Cl_2_, r.t; (b) 1-R-piperazine, N_2_, 1-Pentanol, 135°C.

As shown in Scheme 1, various substituted benzyl chlorides were firstly reacted with piperazine to give **3a-n** (Huang, et al., 2019). Then **3a-n** and **6a-f** were substituted with 2-chloro-4-amino-6,7-dimethoxyquinazoline to give the target compounds **5a-n** and **7a-f** (Scheme 1).

The introduction of active fragments is a common strategy for drug development. Similar in structure to terazosin, Piribedil also has a pyrimidine-2-piperazine group and has been shown to exert anti-PD effects by affecting energy metabolism (Chen et al., 2020). We developed compound **7c** by introducing its unique piperonyl group. The benzofurazan structure, which is a bioisostere with piperonyl, has also been shown to have neuroprotective and anti-PD activities (Barhwal et al., 2009; Swart and Hurley 2016). We introduced this group into new molecules to develop compounds **7b**, **10c** and **12b**. In addition, the tetrazine group, which plays an important role in neuroprotective and cerebrovascular disease drugs, has also been attempted to be integrated into the pyrimidine-2-piperazine group to obtain more potent molecules.

**SCHEME 2:**
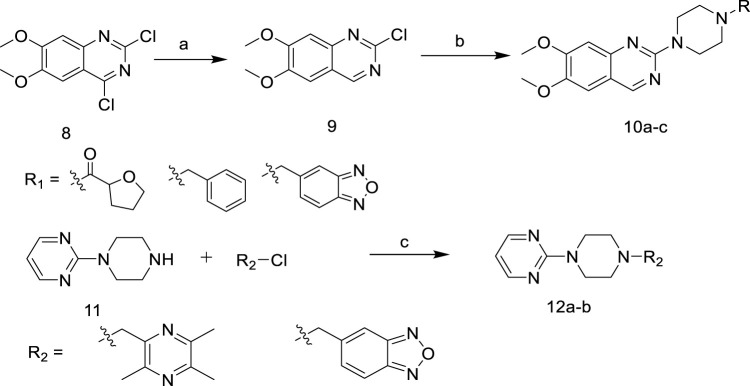
Synthetic of target compounds **10a ∼ c** and **12a ∼ b**. Reagents and conditions: (a) NaBH_4_, EtOH, CH_2_Cl_2_; (b) 1-R-piperazine, N_2_, 1-Pentanol, 135°C; (c) K_2_CO_3_, KI, CH_2_Cl_2_.

In Scheme 2, 2,4-Dichloro-6,7- dimethoxyquinazoline was reduced to obtain intermediate **9**, and then reacted with 1-R-piperazine to obtain the target compounds **10a-c**. In addition, 2-(1-piperazinyl) pyrimidine was reacted with 2-(chloromethyl)-3,5,6-trimethyl- pyrazine or 5-(chloromethyl)-2,1,3-benzoxadiazole to give the target compounds **12a-b**.

Based on the above design, we synthesized a series of terazosin analogs. All the target compounds were structurally characterized by ^1^H NMR, ^13^C NMR and ESI-MS.

### 3.2 Biological Evaluation

Mitochondrial energy metabolism disorder is an essential contributing factor in the degeneration of dopaminergic neurons in Parkinson’s disease, and the SH-SY5Y cell injury model is widely used in neuroprotection research (Zhong et al., 2018). Therefore, all compounds were tested for neuroprotective effects on SH-SY5Y cells induced by MPP^+^, Pgk1 enzyme agonistic activity *in vitro*, and the effects on intracellular ATP level in SH-SY5Y cells induced by MPP^+^. The results are summarized in Table 1, Table 2, and Table 3. In addition, compounds **7d**, **10a**, **12a** and **12b** were studied on the ROS level of SH-SY5Y cells induced by MPP^+^. The results are summarized in Figure 2.

**TABLE 1:** The survival rate of SH-SY5Y cells induced by MPP^+^.

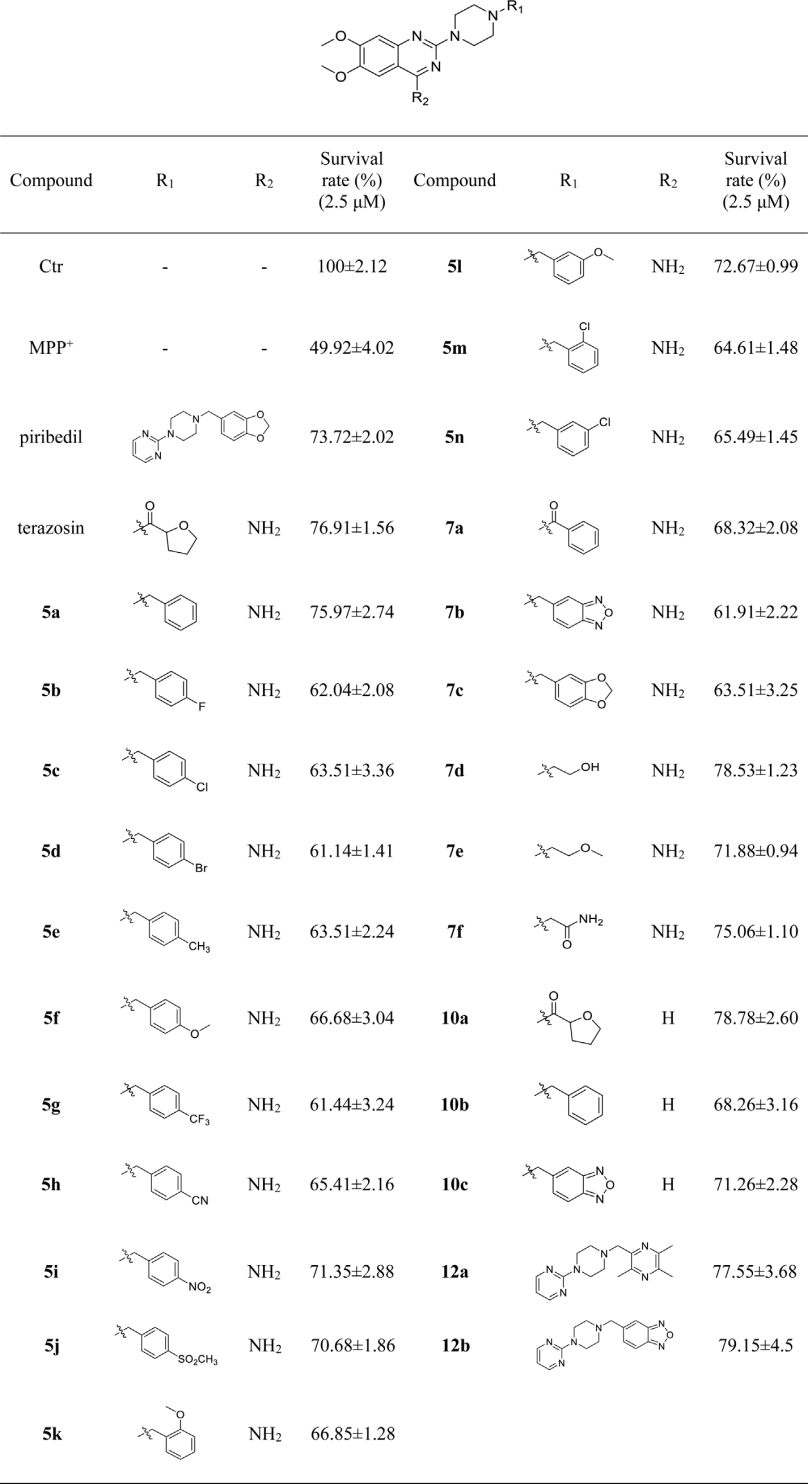

**TABLE 2:** Effect of target compounds on Pgk1 activity *in vitro*.

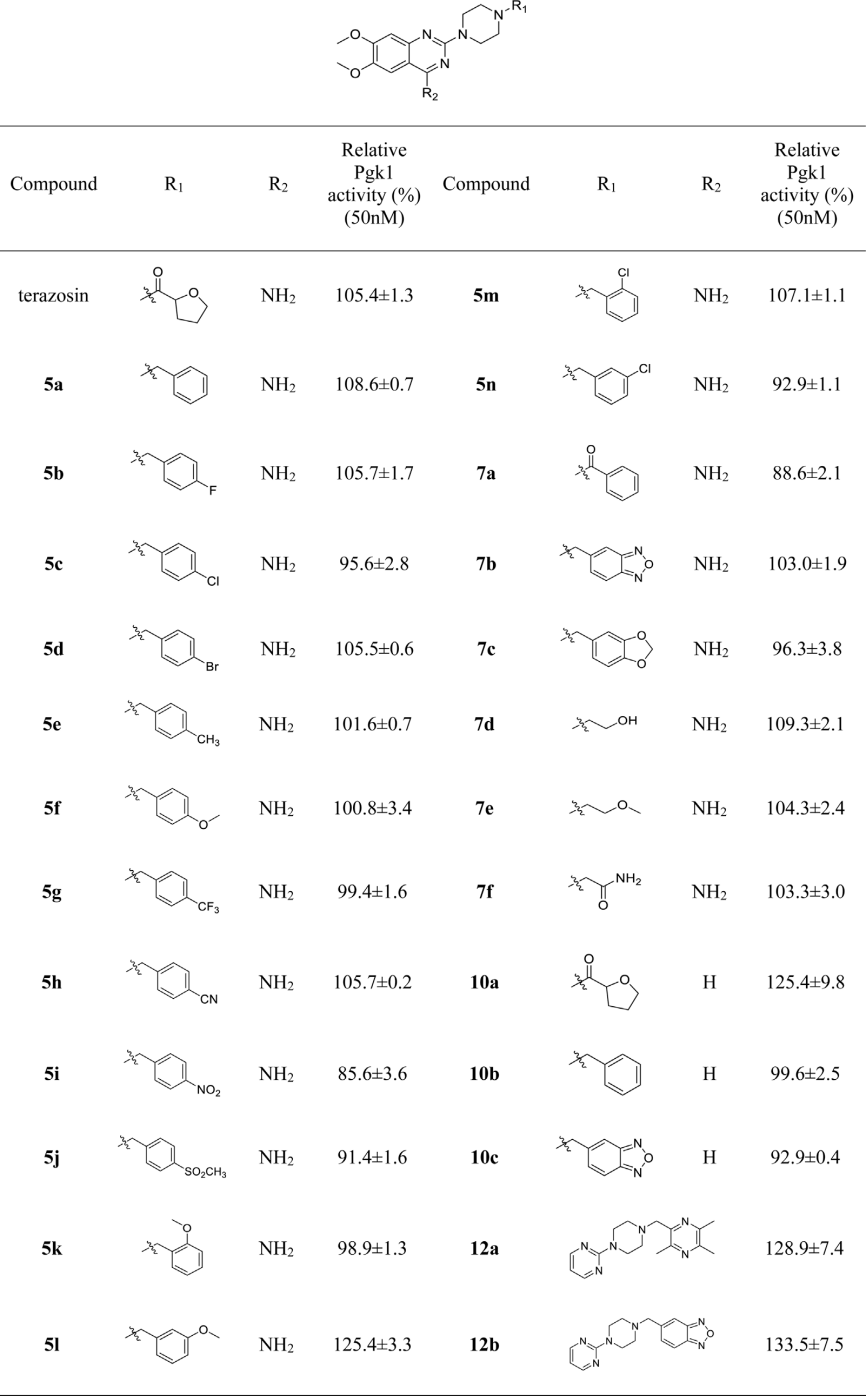

**TABLE 3:** Relative ATP levels in SH-SY5Y nerve cells induced by MPP^+^ by the target compound at a concentration of 2.5 μM.

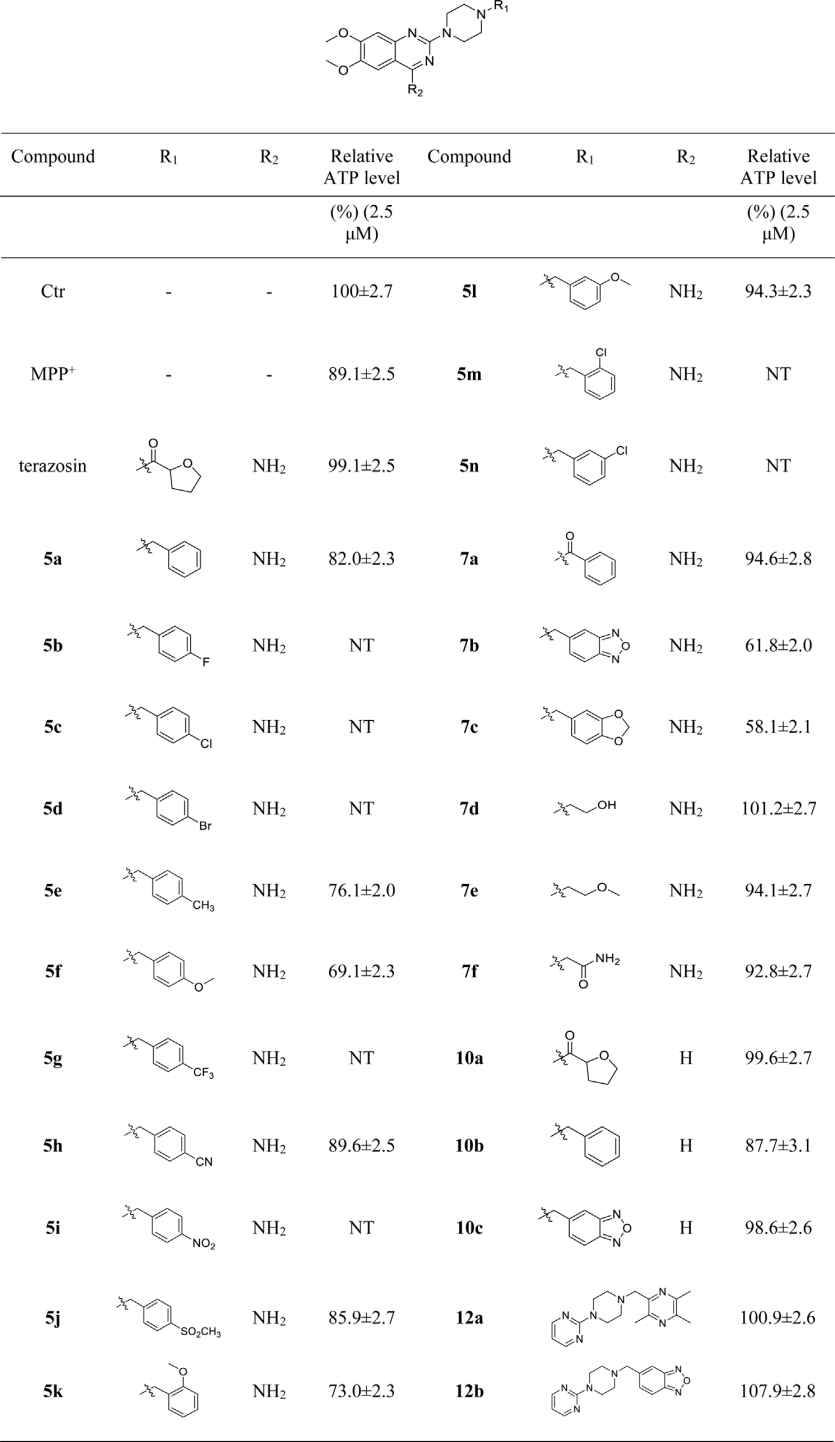

#### 3.2.1 Neuroprotective Effects of Target Compounds *In Vitro*

We first evaluated the neuroprotective activity of all target compounds through the MPP^+^ induced SH-SY5Y cell injury model. Terazosin and piribedil were used as positive controls. Dosing concentration refers to the test results of terazosin (Supplementary Figure S26), and is uniformly set to 2.5 μM. The results are shown in Table 1. Almost all terazosin analogs showed protective effects. Among them, the neuroprotective activities of compounds **7d**, **10a**, **12a** and **12b** are close to or stronger than those of the control drugs.

#### 3.2.2 The Agonistic Activity of Target Compounds on Pgk1 *In Vitro*

Referring to the reported optimal activation concentration of terazosin on Pgk1, we evaluated the agonistic activity of all terazosin analogs on Pgk1 *in vitro*, terazosin was used as a positive control. The results are shown in Table 2. At the concentration of 50 nM, some terazosin analogs showed Pgk1 agonistic activity. Among them, **5l**, **10a**, **12a** and **12b** are significantly stronger than the others, even better than the control drug.

**FIGURE 3:**
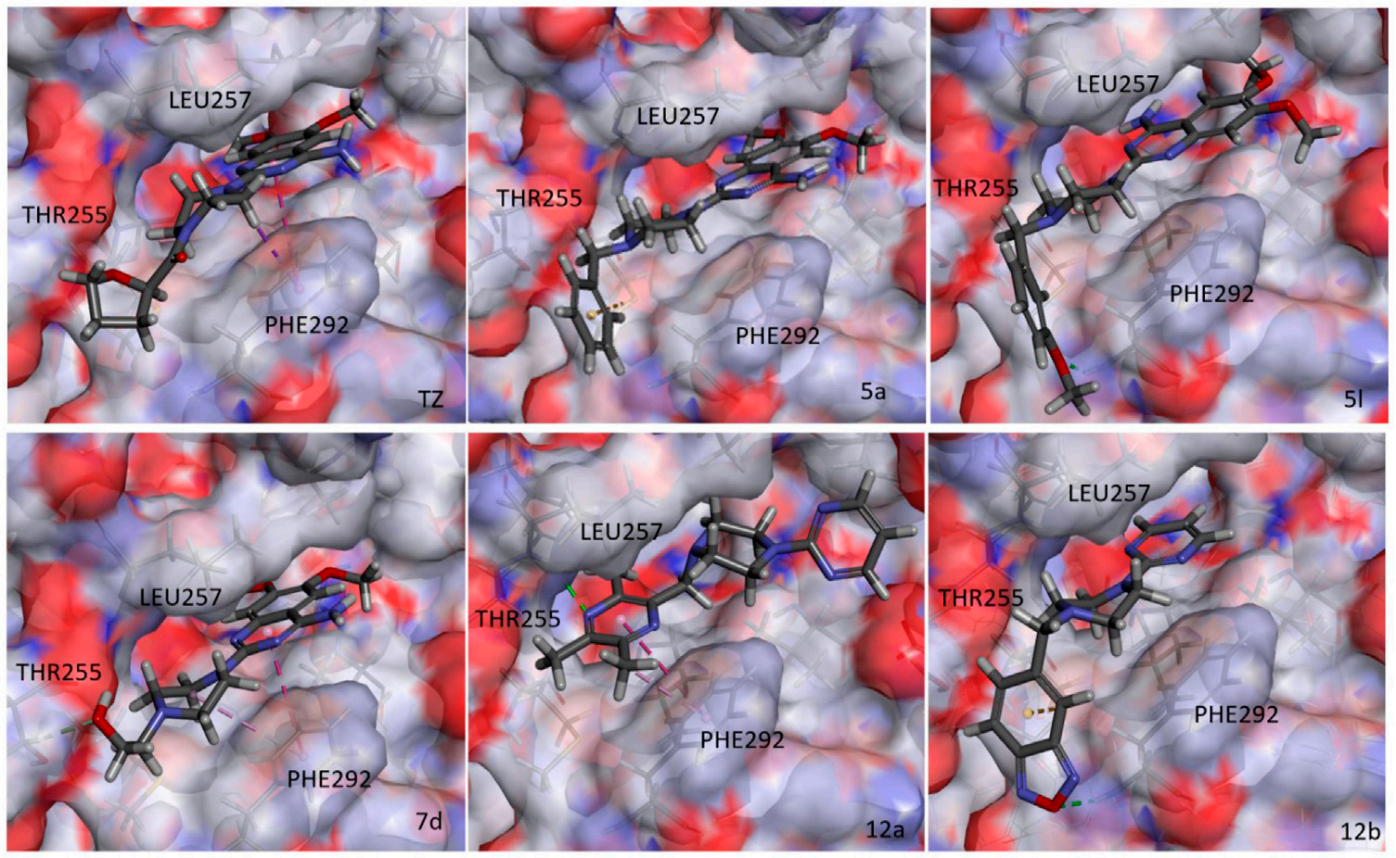
Docking study of terazosin analogs and Pgk1 (PDB ID: 4O3F).

The results of the above two experiments showed that terazosin analogs exhibited protective effects on nerve cell injury induced by MPP^+^, and many terazosin analogs exhibited agonistic effects on Pgk1. There was a significant correlation between enzyme activity and cell activity. Based on these findings, we analyzed and discussed the SAR of terazosin analogs. When the R group at the C-2 position is substituted with benzyl, the activity of the target compounds is decreased. The electron-withdrawing and electron-donating groups on the benzene ring will further reduce the neuroprotective activity and the Pgk1 agonistic activity. This may be due to the lack of interaction with the residue of Pgk1, thereby reducing the affinity. However, the activity of **5l** is slightly better. This is probably due to the re-established hydrogen bond interaction between the methoxy group and the Phe292 residue of Pgk1 (Figure 3). The poor activity of compounds **7b** and **7c** is probably due to the large steric hindrance of the benzofurazan and piperonyl groups, preventing them from entering the active pocket. Compounds **7d** ∼ **7f** are ring-opening derivatives of the terazosin, which can better fit the active pocket of Pgk1 than the benzene ring. Among them, the hydroxyl group of **7d** can form a hydrogen bond interaction with the Thr255 residue of Pgk1, increasing its activity. Compound **10a** is the deamination product of terazosin, and its activity is equivalent to that of the original drug, indicating that the amino group on quinazoline is not essential for activity. The **12a** and **12b** are fusion products of terazosin and piribedil backbones. Although they reach into the pocket clefts are shallower than terazosin, key interactions are maintained. **12a** has a hydrogen-bonding interaction with the Leu257 residue of Pgk1, and compound **12b** has a hydrogen-bonding interaction with the Phe292 residue of Pgk1, which enhances the binding between them and Pgk1. Thus, improving their activity. In view of the best neuroprotective activity and Pgk1 agonism of compound **12b**, we further studied its mechanism. Other compounds with good activity were also tested.

#### 3.2.3 Effects of Target Compounds on Intracellular ATP Content of SH-SY5Y Cells Induced by MPP^+^

Intracellular ATP level is essential for cell survival, and Pgk1 has the function of promoting ATP production, which helps to maintain the function and survival of nerve cells. Based on the above factors, we evaluated the effects of terazosin analogs on the ATP levels in SH-SY5Y cells induced by MPP^+^. Terazosin was used as a positive control. The results are shown in Table 3. The effects of compounds **7d**, **10a**, **12a** and **12b** significantly increase ATP levels in SH-SY5Y cells induced by MPP^+^ at a concentration of 2.5 μM. This is consistent with their neuroprotective effect.

#### 3.2.4 Effects of Target Compounds on ROS Level of SH-SY5Y Cells Induced by MPP^+^

**FIGURE 4:**
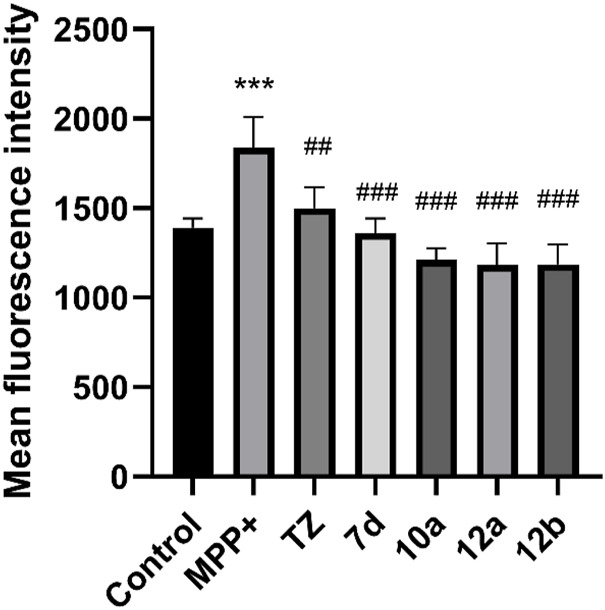
Effect of target compounds on ROS level of SH-SY5Y cells injured by MPP^+^. Compounds were all at the concentration of 2.5 μM and were tested under the same conditions. Values are means ± SD. Statistical significance was represented by ^***^ *p* < 0.001 *vs*. control, ^##^ *p* < 0.01 *vs*. MPP^+^ group, ^###^ *p* < 0.001 *vs*. MPP^+^ group.

Reactive oxygen species (ROS) is another key factor in cell injury. High levels of reactive oxygen species will destroy the function of DNA and mitochondria, resulting in nerve cell death and loss of function. Finally, we evaluated the effects of compounds **7d**, **10a**, **12a** and **12b** on ROS levels in SH-SY5Y cells induced by MPP^+^. As shown in Figure 4, compounds **7d**, **10a**, **12a** and **12b** can reduce ROS levels in SH-SY5Y cells induced by MPP^+^ at a concentration of 2.5 μM, and the effect is stronger than terazosin. This shows that reducing the level of ROS is also one of the mechanisms of terazosin analogs to play a neuroprotective role. Moreover, the ability to reduce reactive oxygen species is related to the survival of nerve cells and Pgk1 activity.

## 4 Conclusion

We designed a series of terazosin analogs with 6,7-dimethoxy-2-(piperazin-1-yl)-quinazoline as backbone, and synthesized **12a** and **12b** by backbone fusion. Among them, the more active compounds **7d**, **10a**, **12a**, and **12b** can promote glycolysis by activating Pgk1, thereby increasing the intracellular ATP content, reducing the ROS level, and playing a neuroprotective role. In summary, based on the co-crystal structure of terazosin and Pgk1, and the working principle of the receptor protein, we developed a series of nitrogen-containing heterocyclic compounds targeting Pgk1 and elucidated the mechanism of its neuroprotective effect. This study further supports the view that reduced ATP content and oxidative stress damage may play an essential role in the pathophysiological mechanism underlying PD and provides a reference for the use of nitrogen-containing heterocyclic compounds for neuroprotection and anti-PD therapy.

## Data Availability

Publicly available datasets were analyzed in this study. This data can be found here: https://www.rcsb.org/structure/2X15 and https://www.rcsb.org/structure/4O3F.
