# Supplementary material for: Terazosin Analogs Targeting Pgk1 as Neuroprotective Agents: Design, Synthesis, and Evaluation
Source: Front Chem. 2022 Jul 26;10:906974. doi: 10.3389/fchem.2022.906974 (PMC9360532; doi:10.3389/fchem.2022.906974)
Supplement: Supplementary file 1 [file DataSheet1.docx]

Supplementary Material

# ^1^H NMR and ^13^C NMR

**
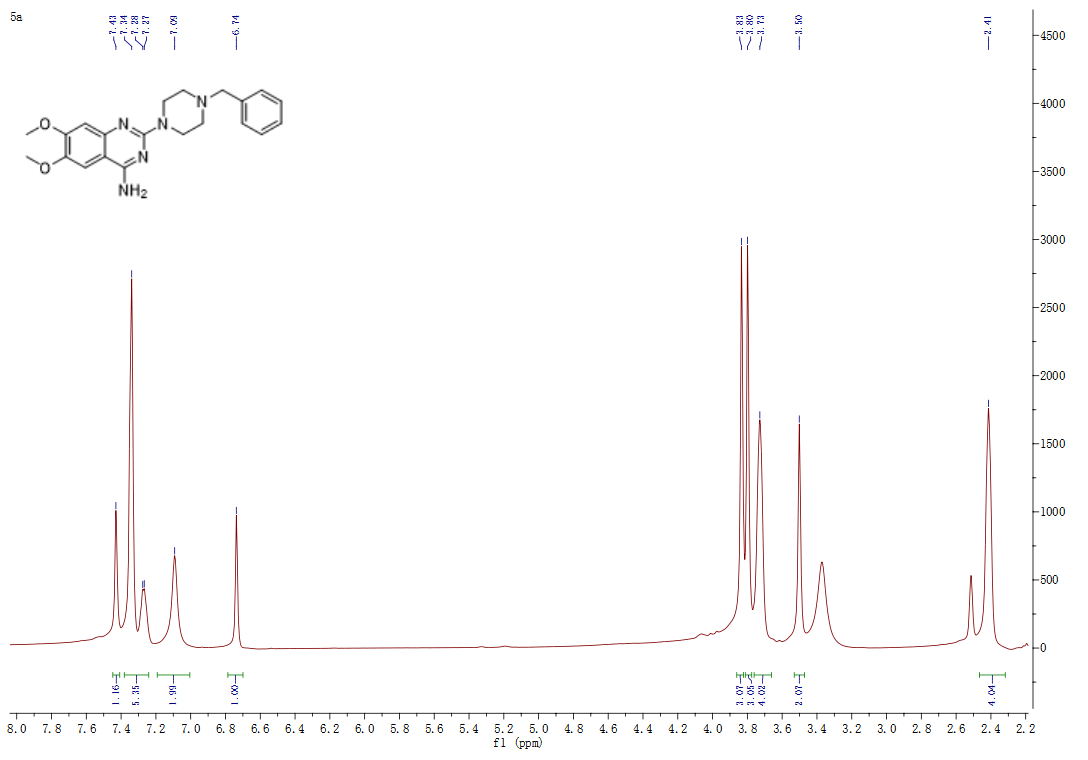
**

**
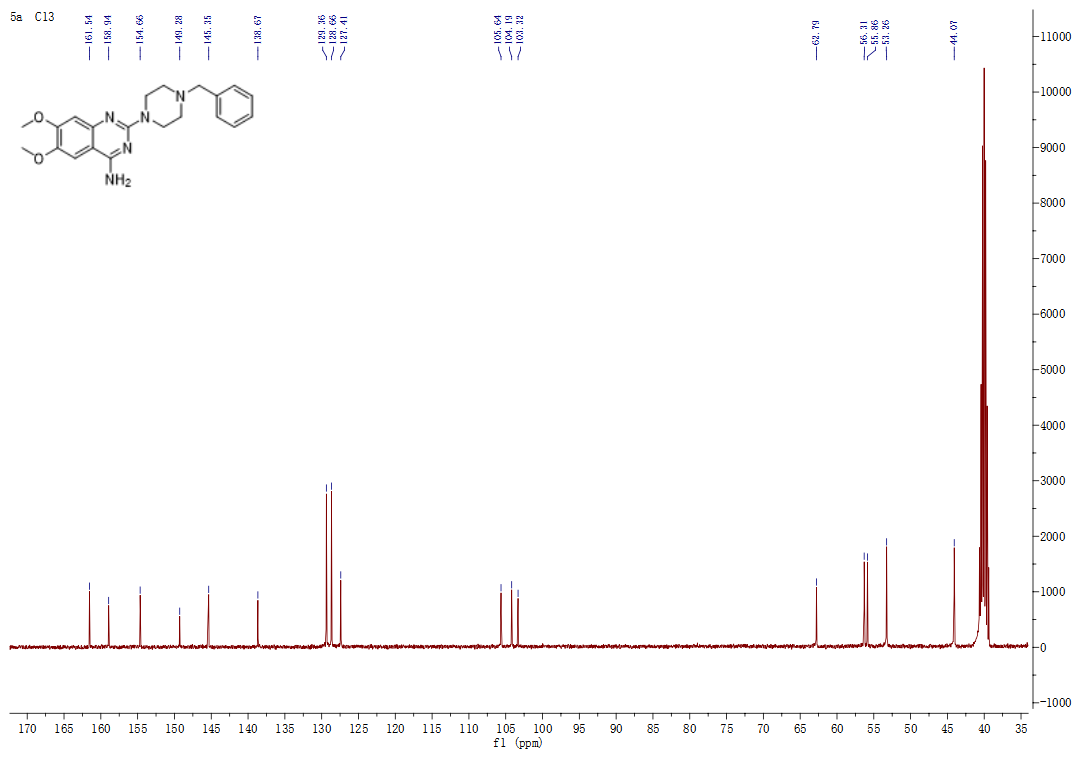
**

**Figure S1.** NMR spectrum of compound (**5a**)

**
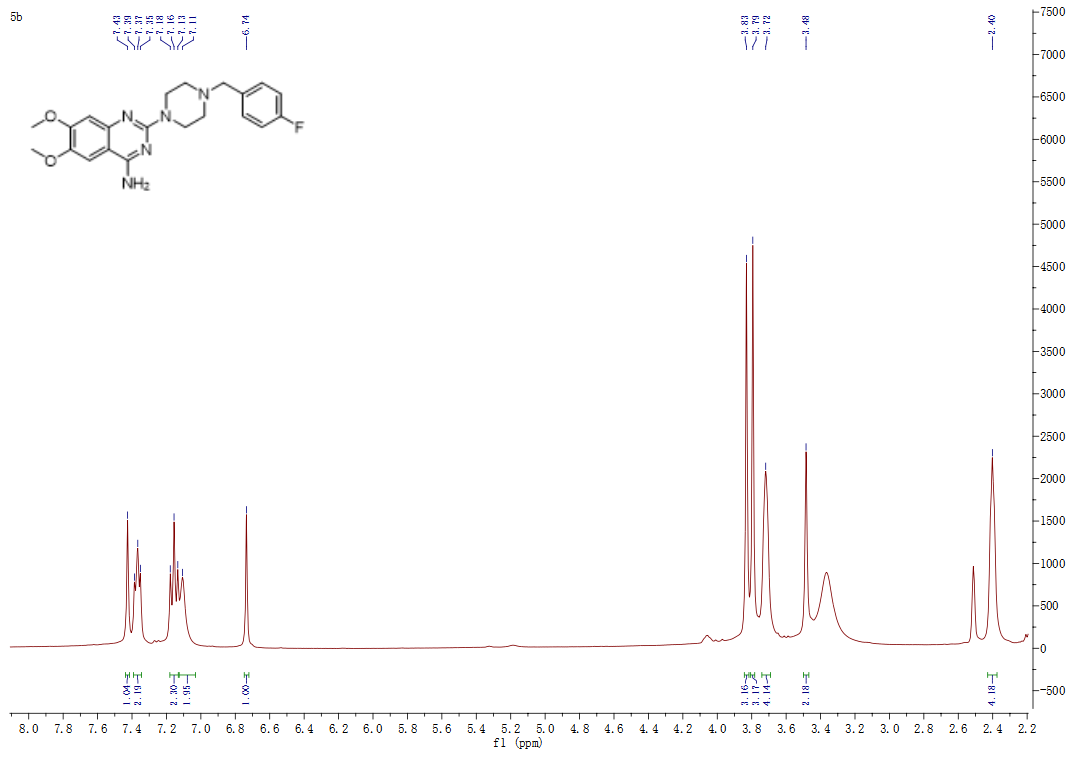
**

**
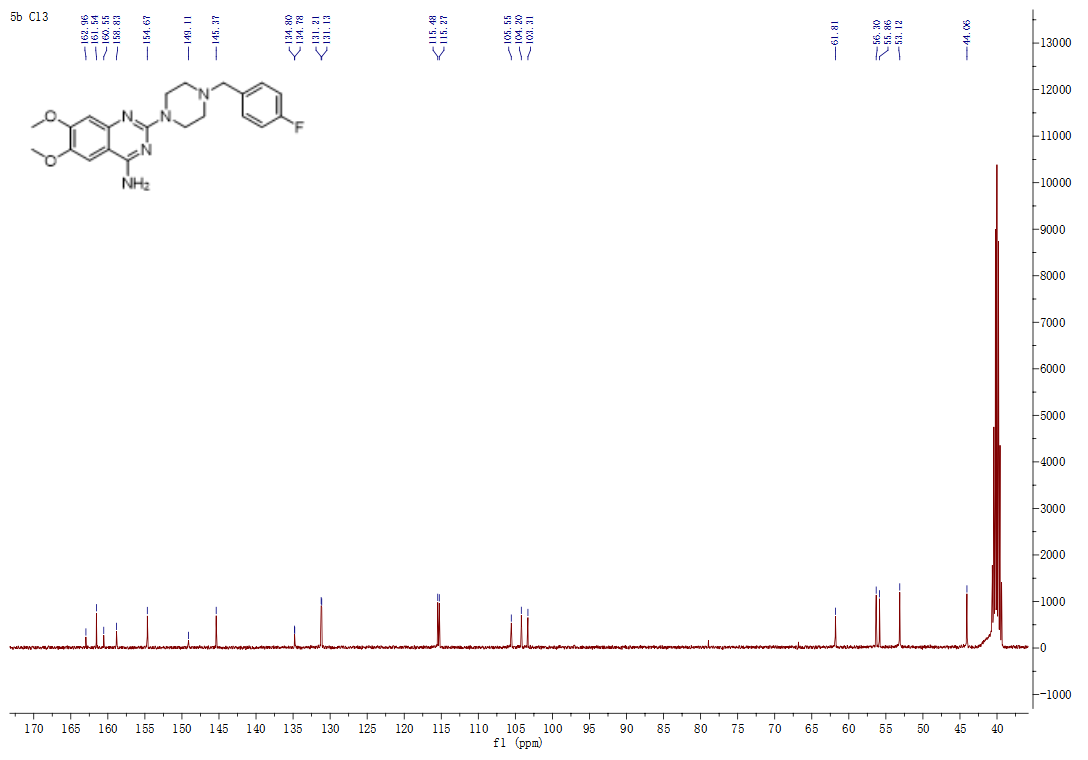
**

**Figure S2.** NMR spectrum of compound (**5b**)

**
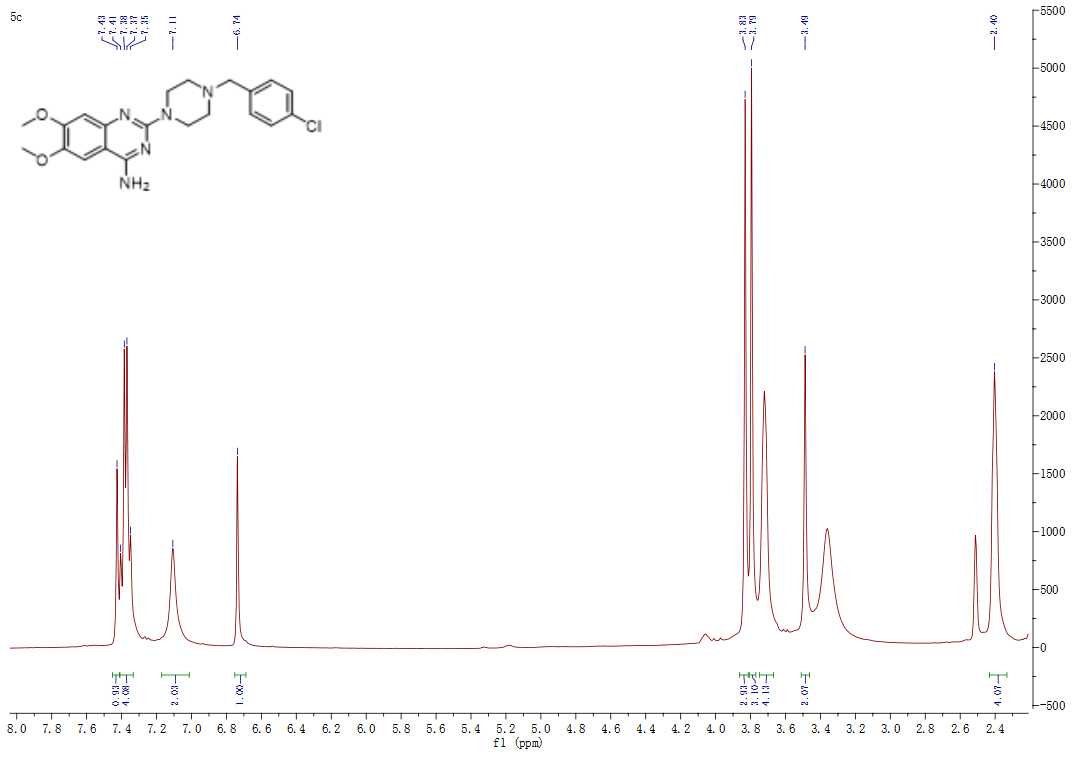
**

**
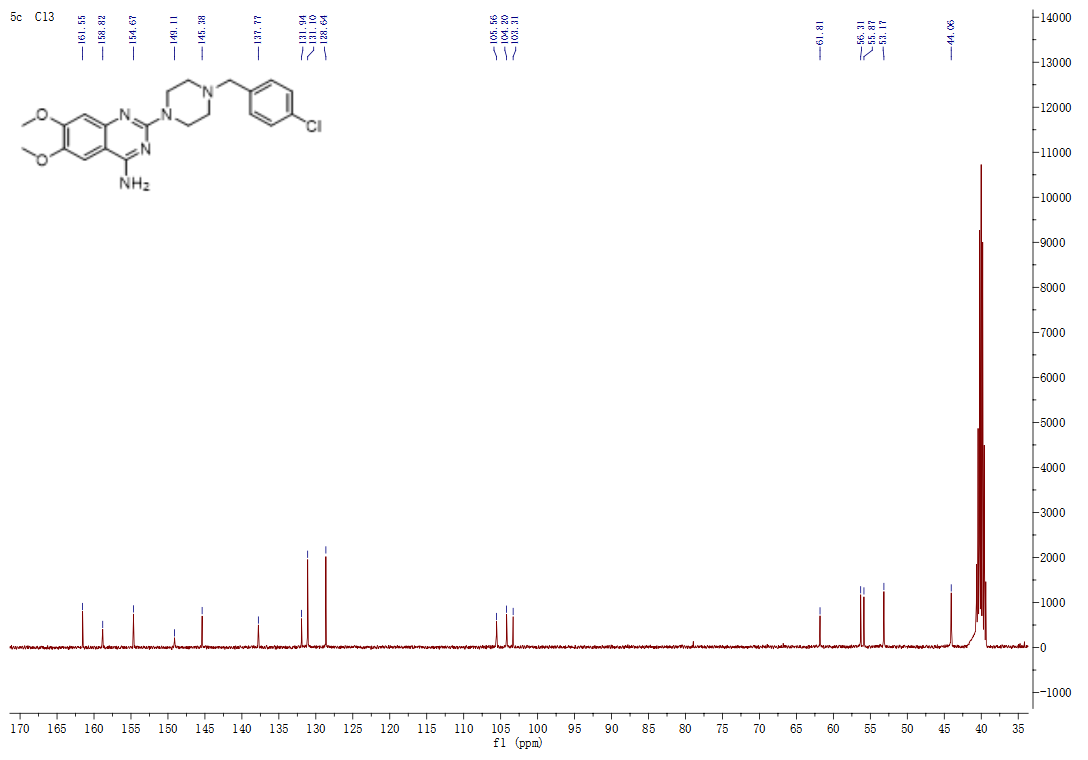
**

**Figure S3.** NMR spectrum of compound (**5c**)

**
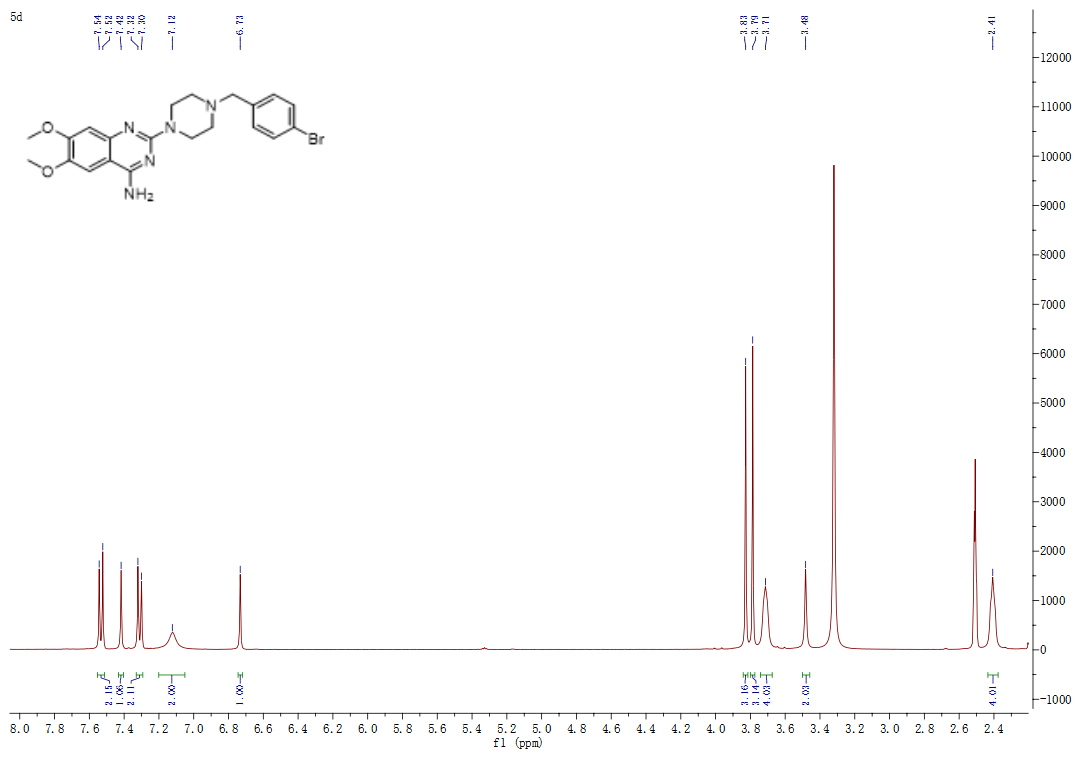
**

**
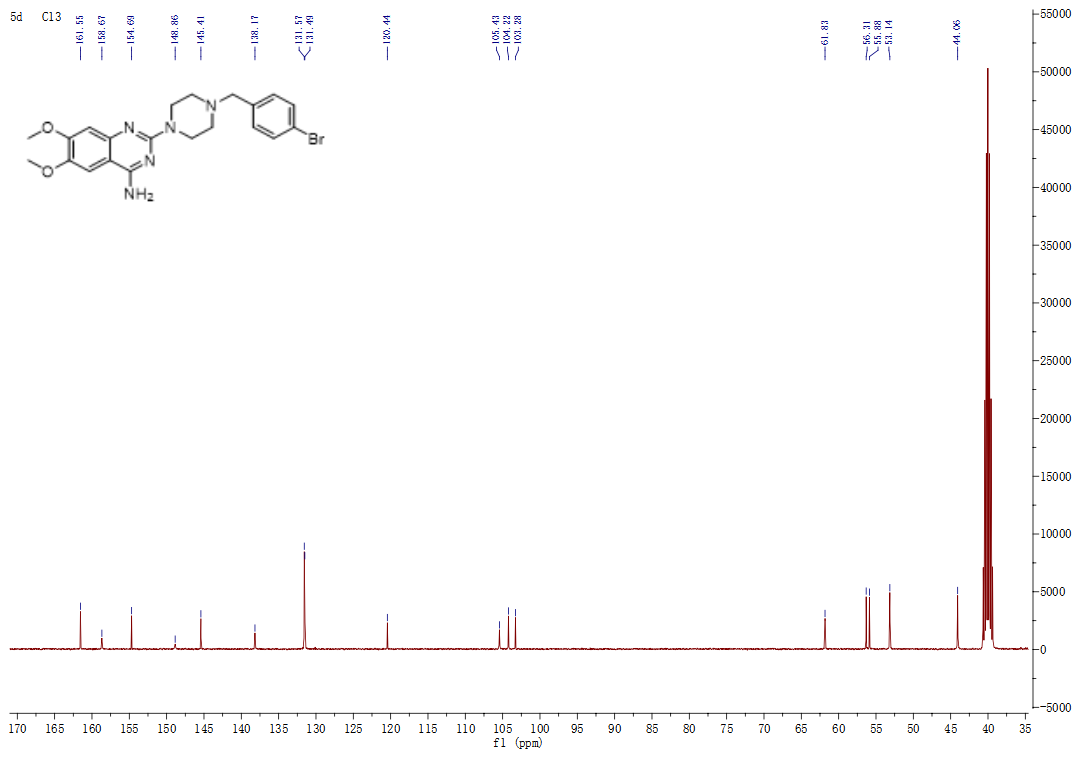
**

**Figure S4.** NMR spectrum of compound (**5d**)

**
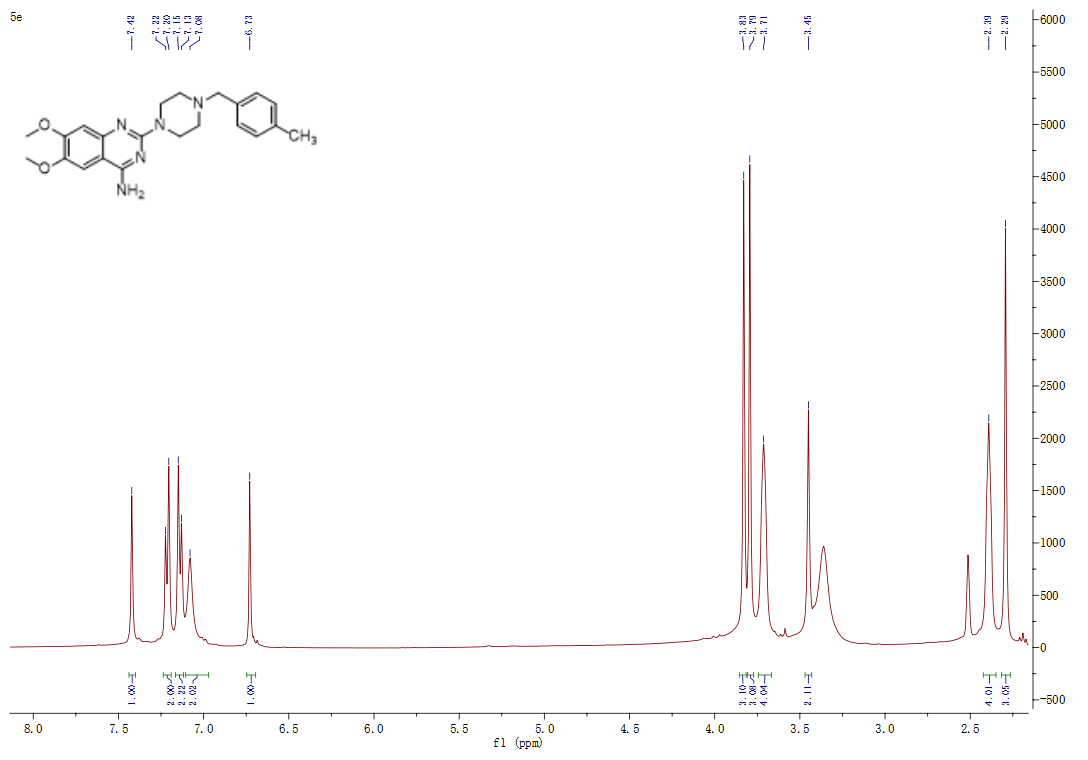
**

**
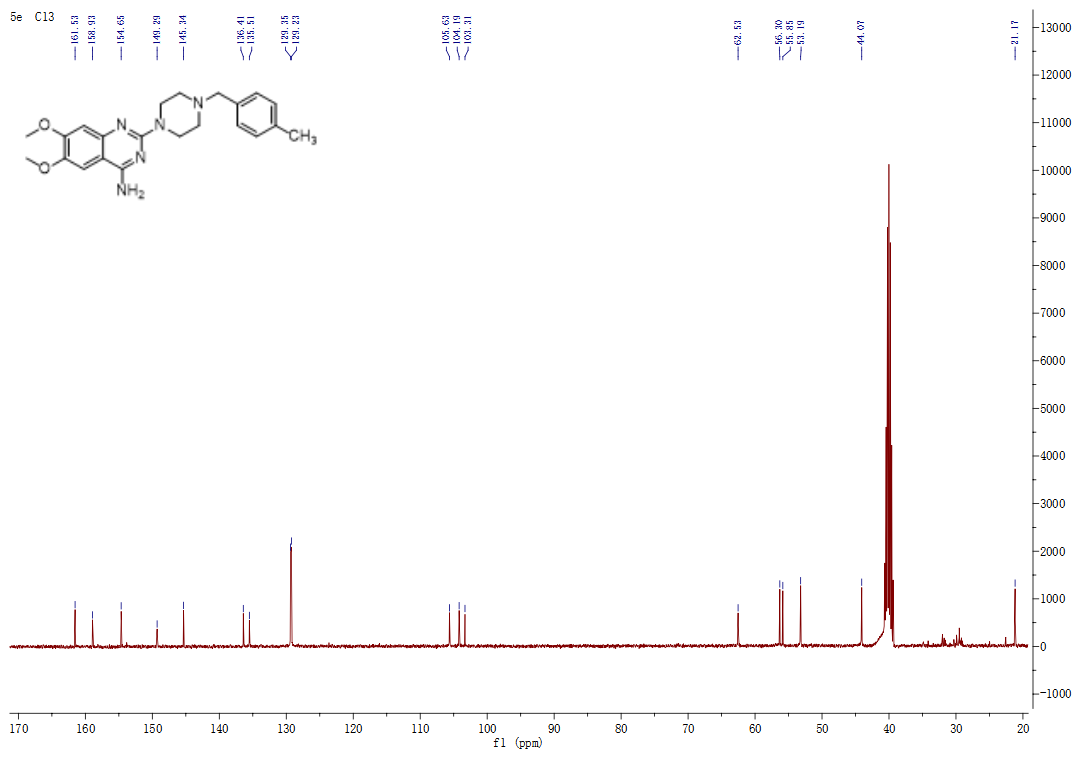
**

**Figure S5.** NMR spectrum of compound (**5e**)

**
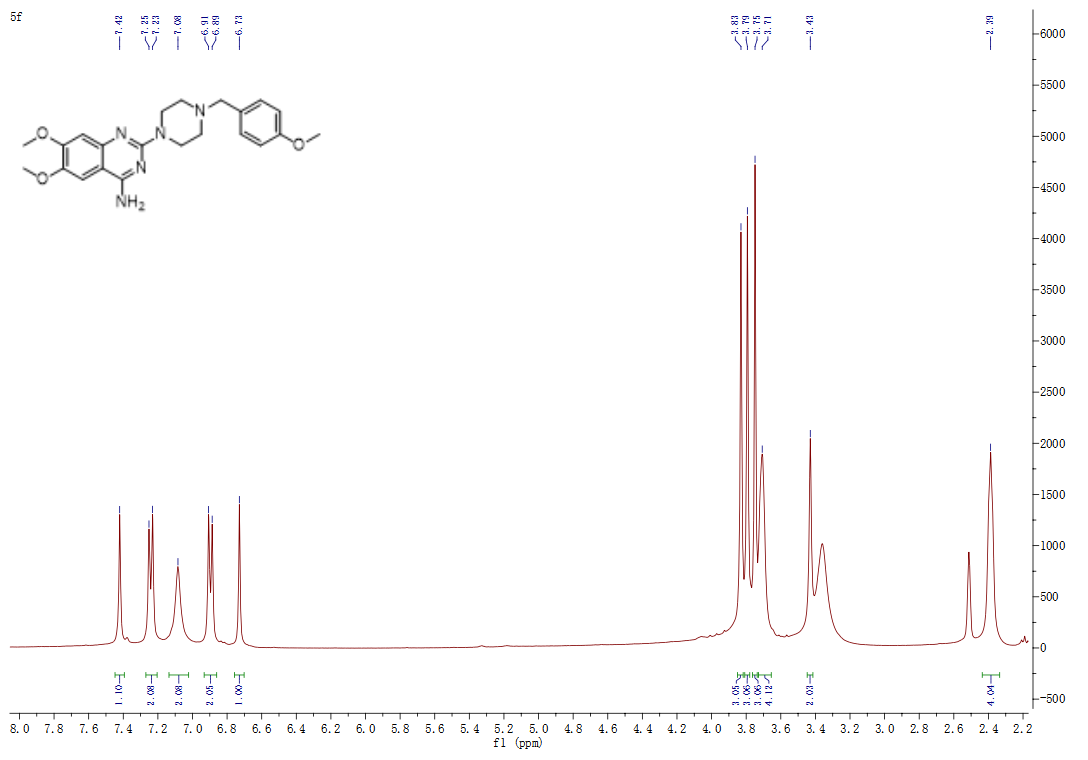
**

**
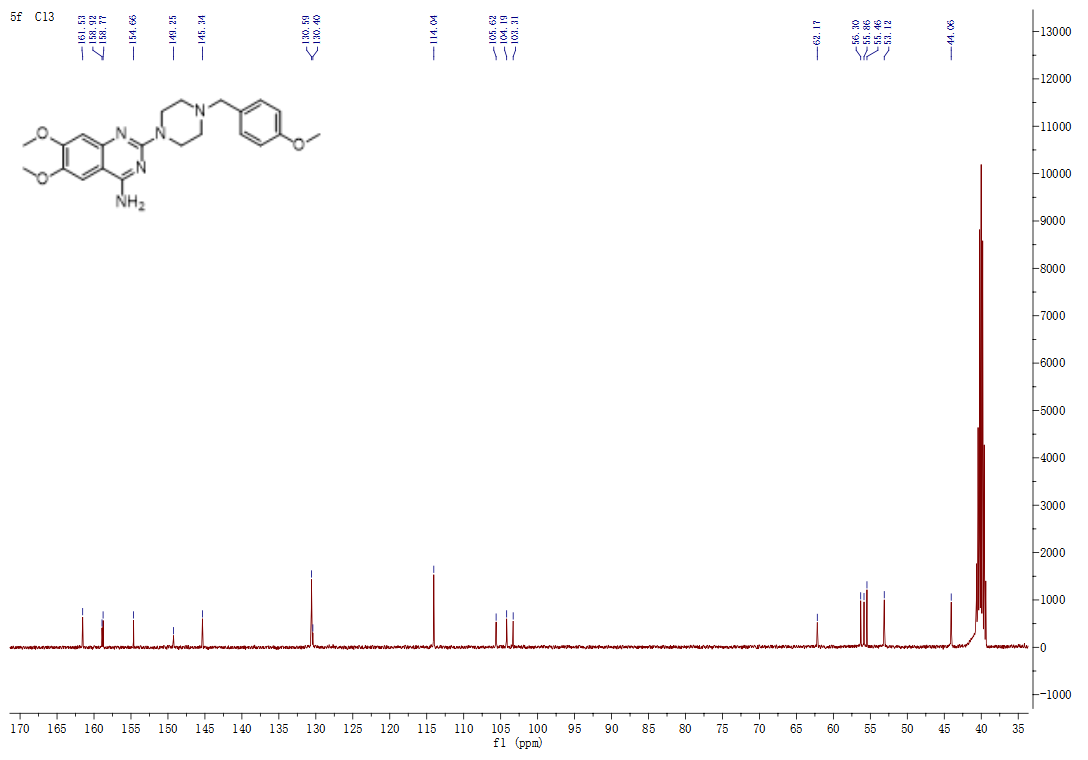
**

**Figure S6.** NMR spectrum of compound (**5f**)

**
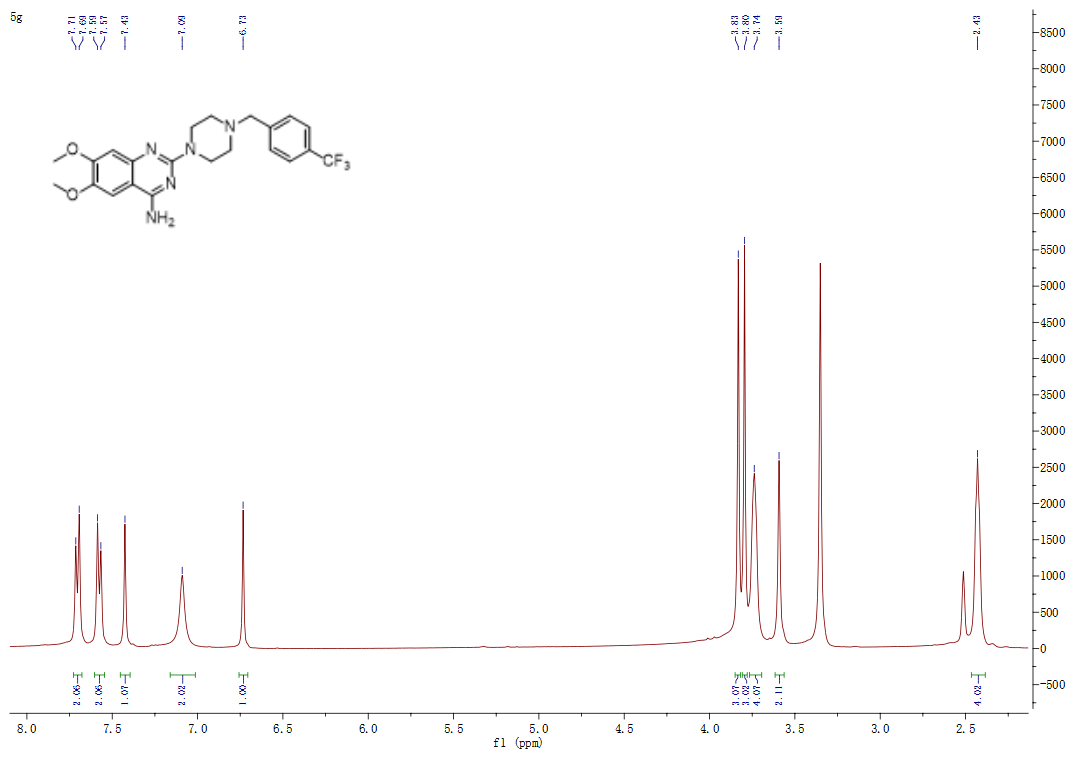
**

**
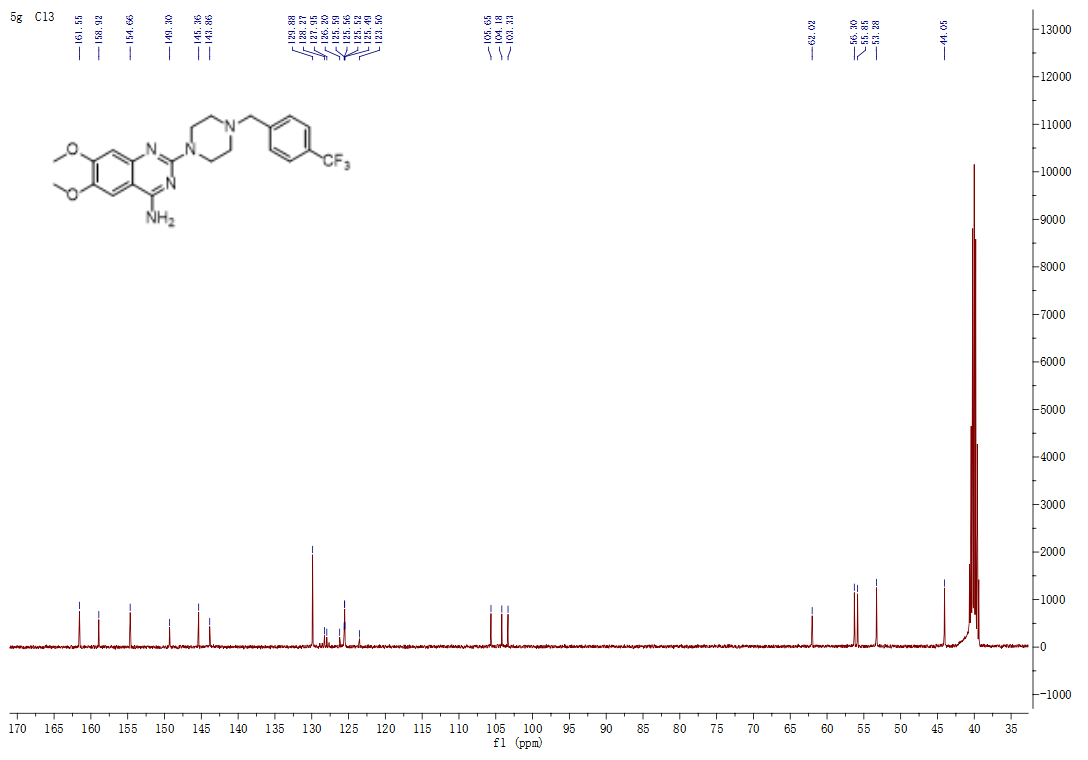
**

**Figure S7.** NMR spectrum of compound (**5g**)

**
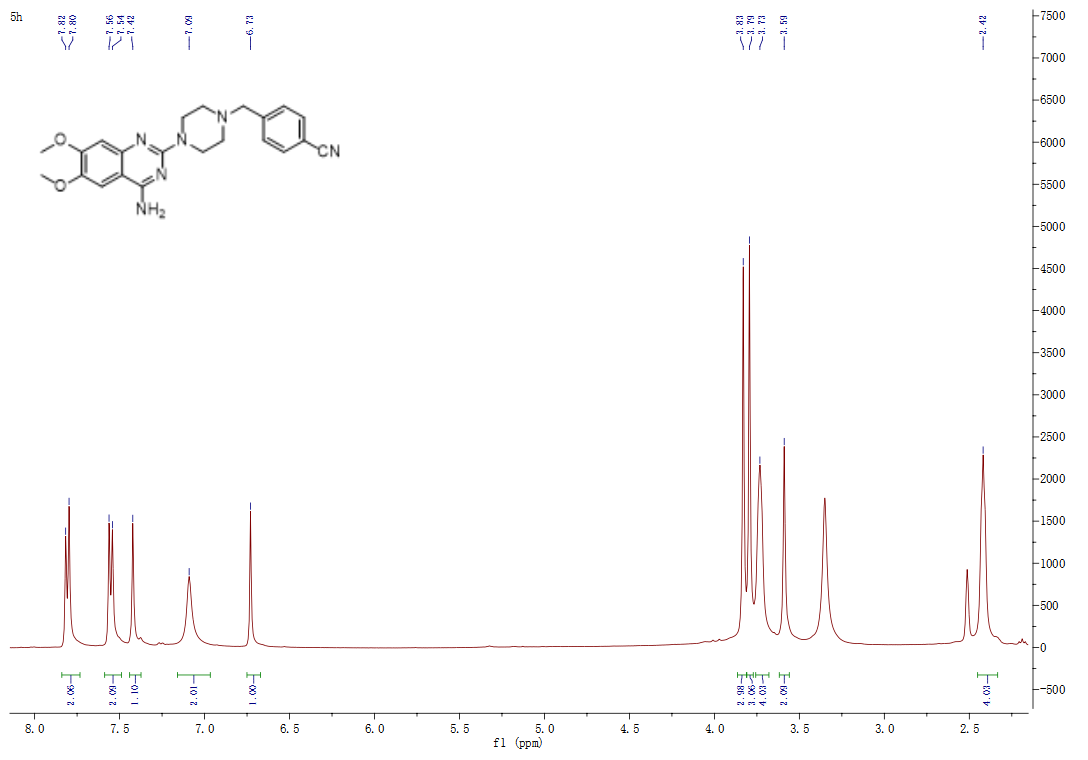
**

**
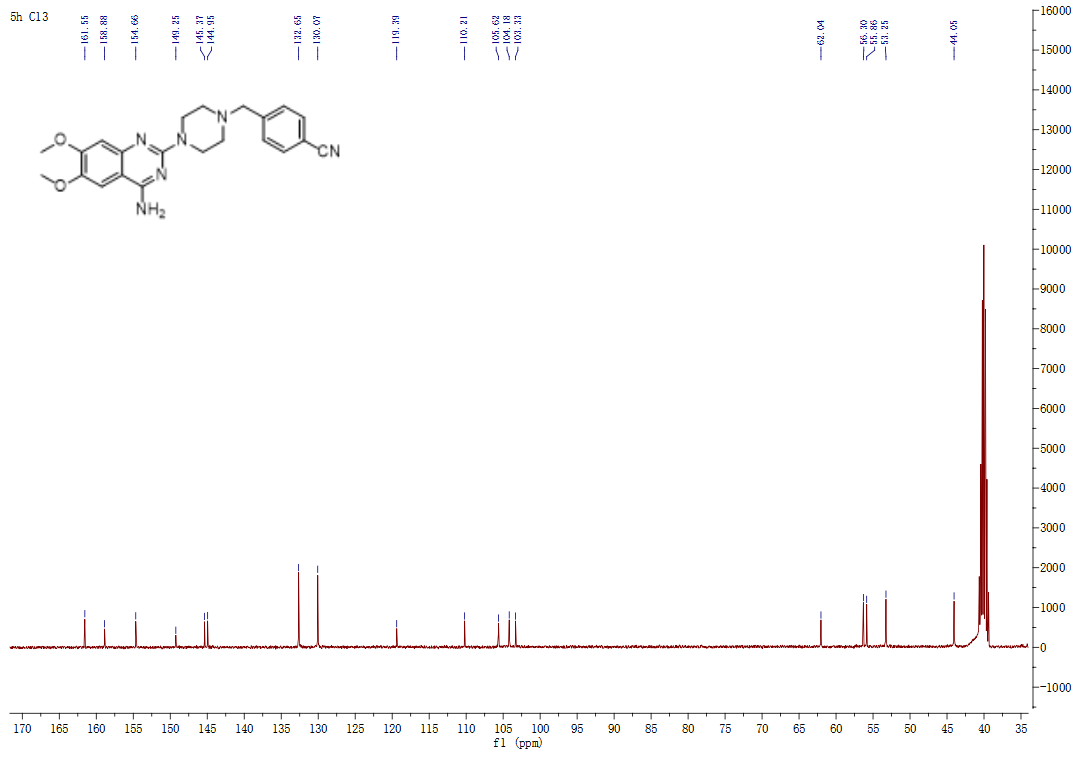
**

**Figure S8.** NMR spectrum of compound (**5h**)

**
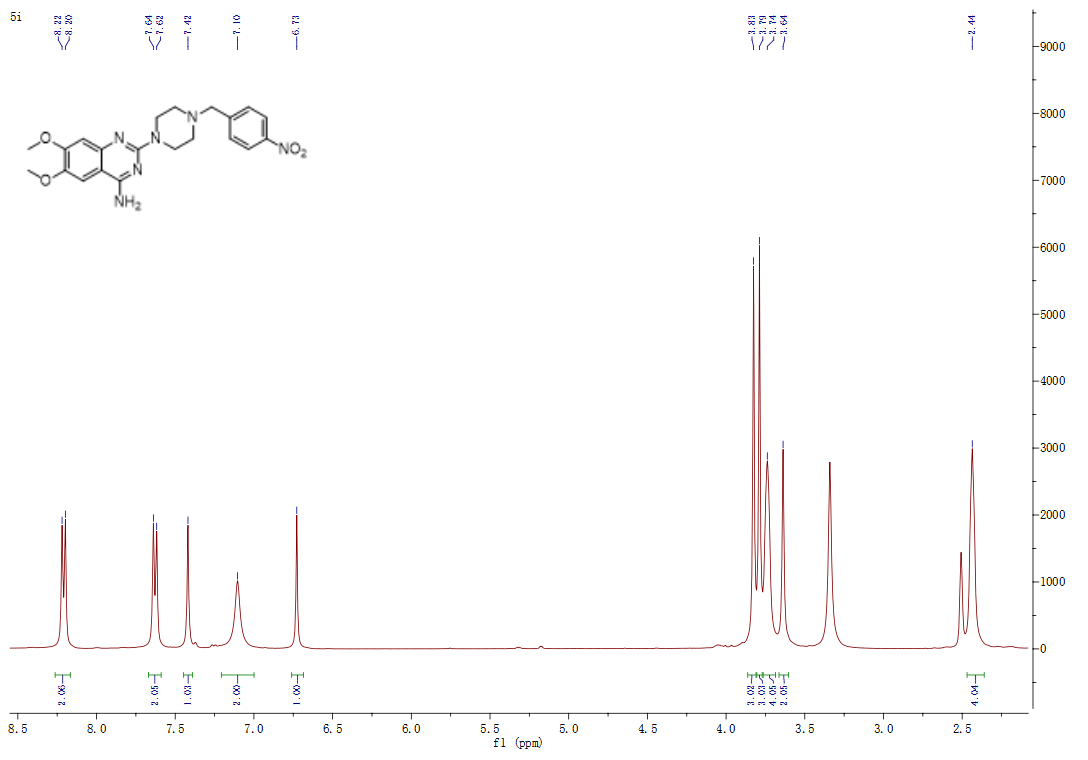
**

**
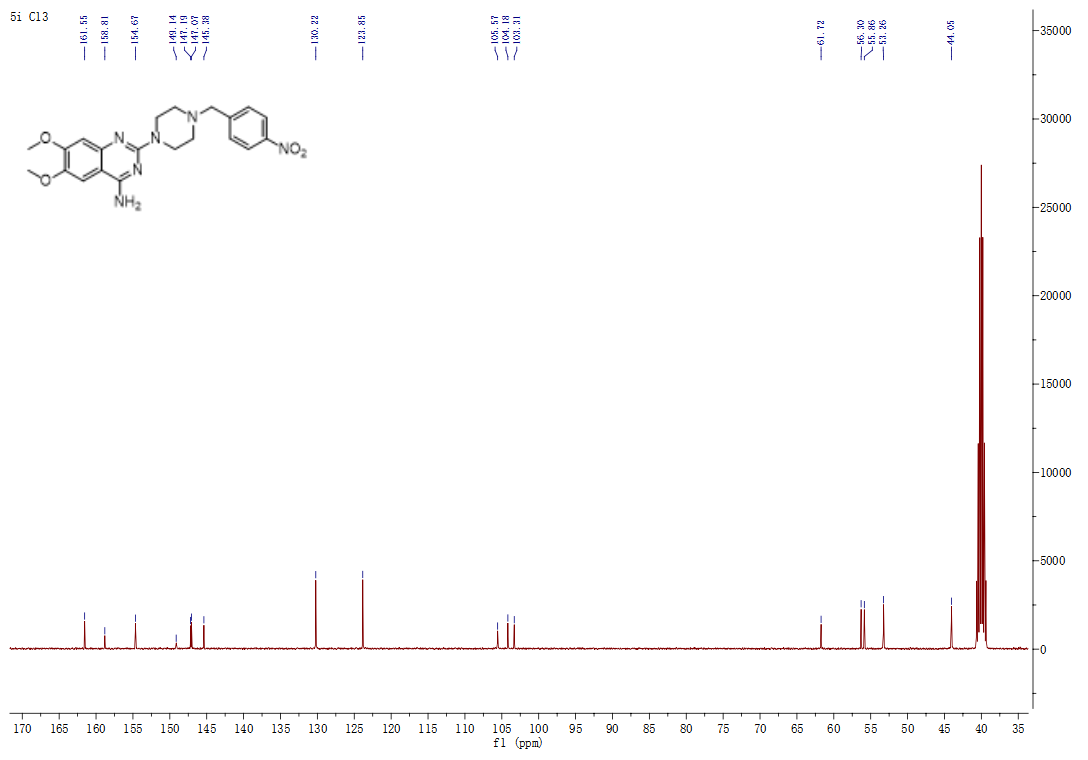
**

**Figure S9.** NMR spectrum of compound (**5i**)

**
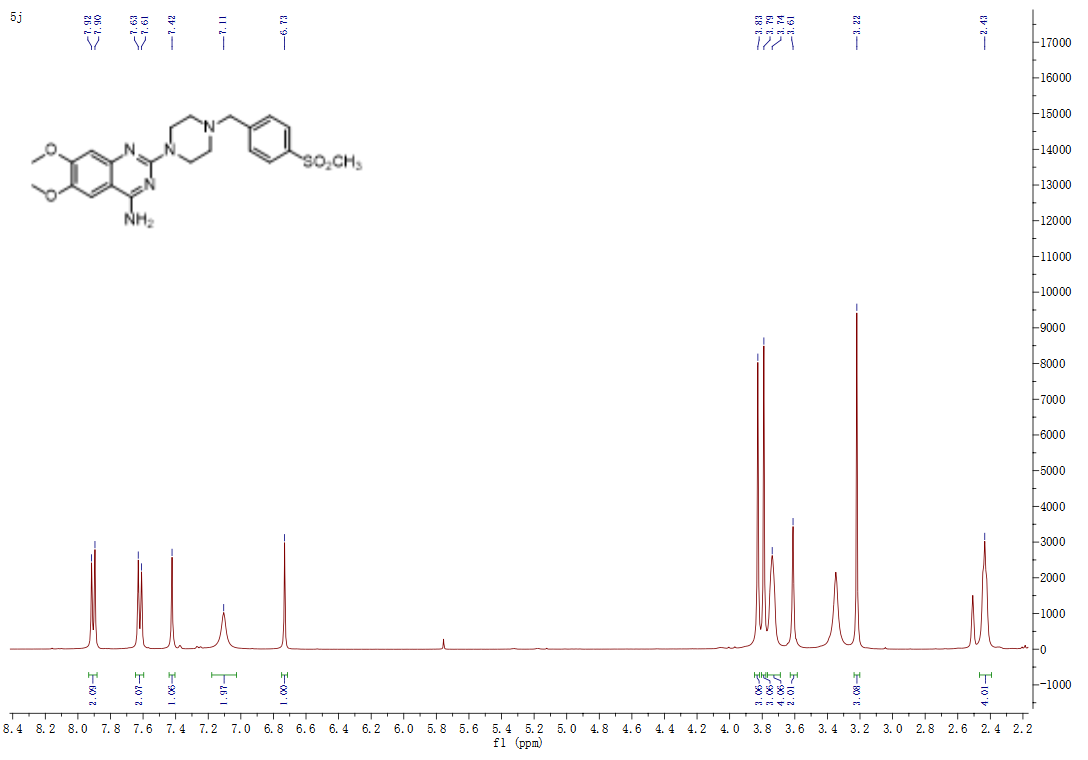
**

**
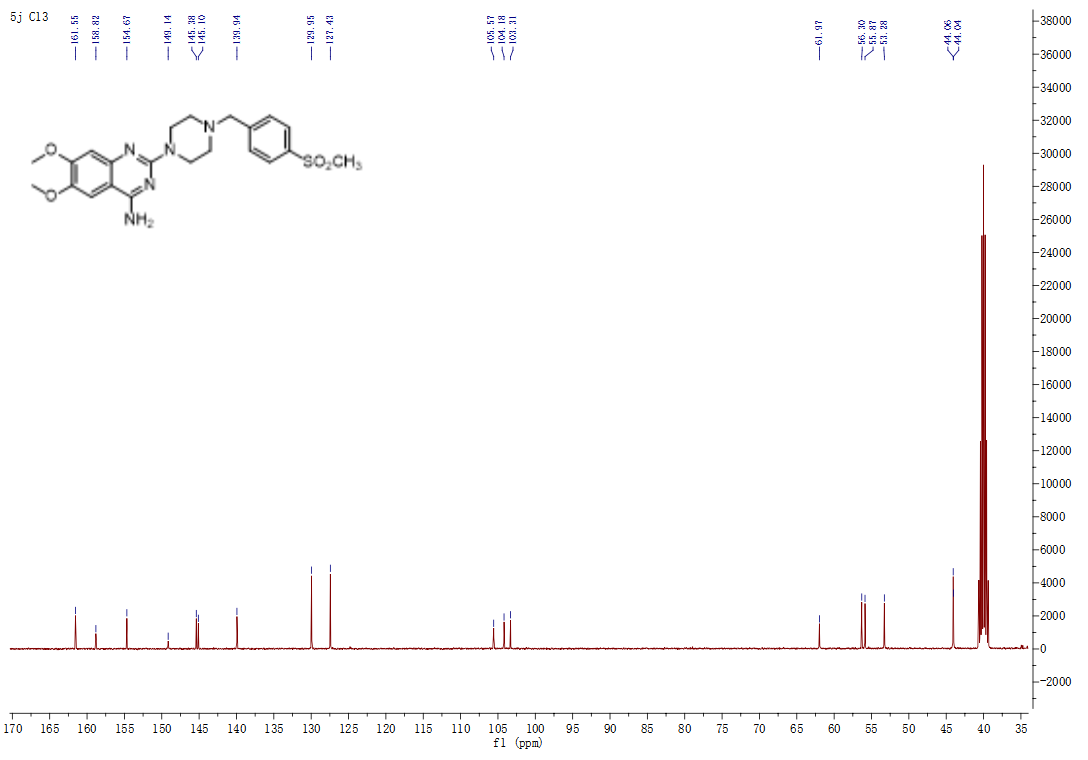
**

**Figure S10.** NMR spectrum of compound (**5j**)

**
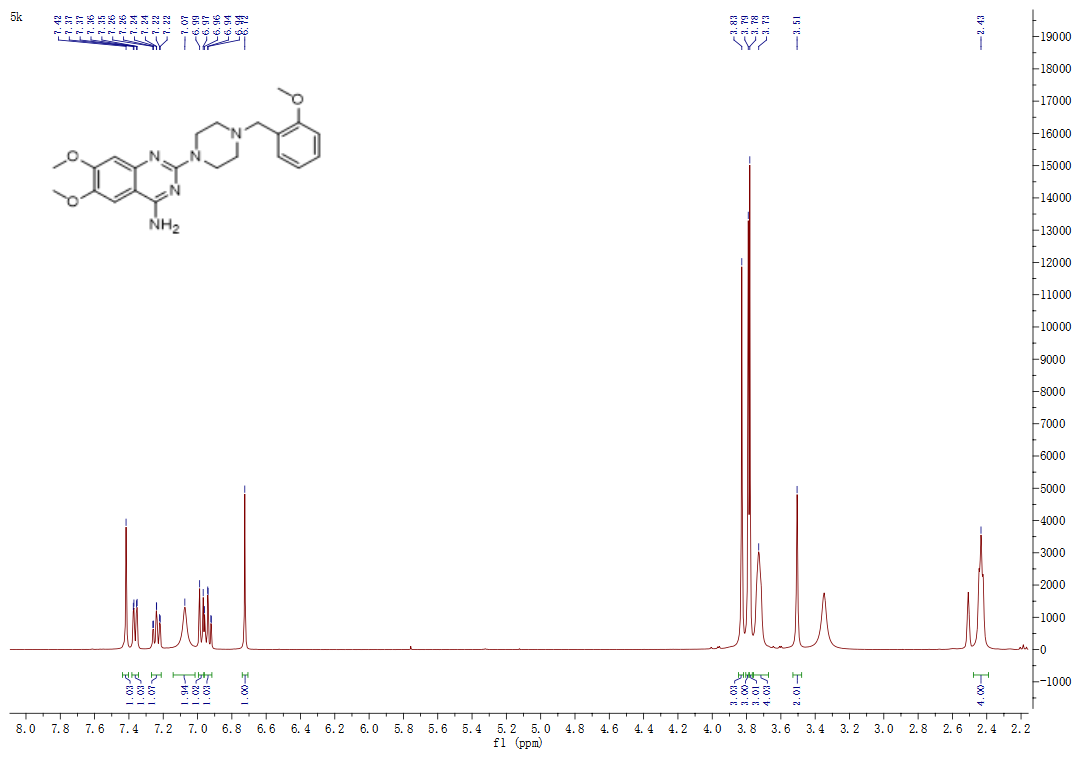
**

**
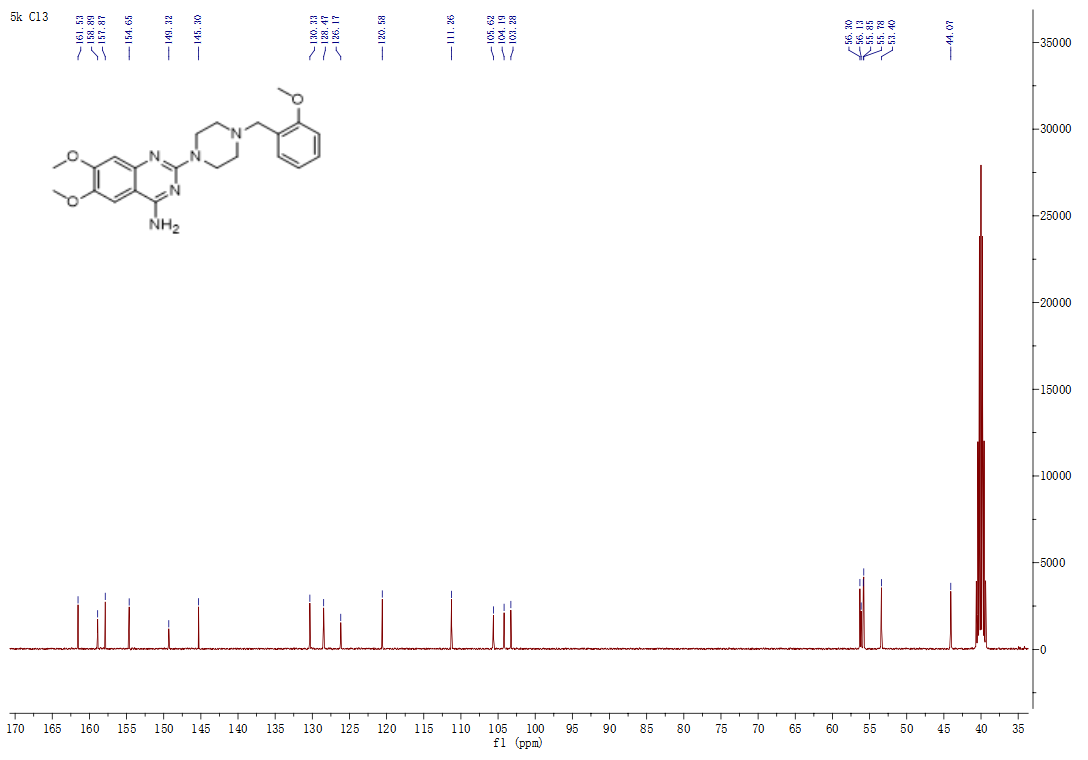
**

**Figure S11.** NMR spectrum of compound (**5k**)

**
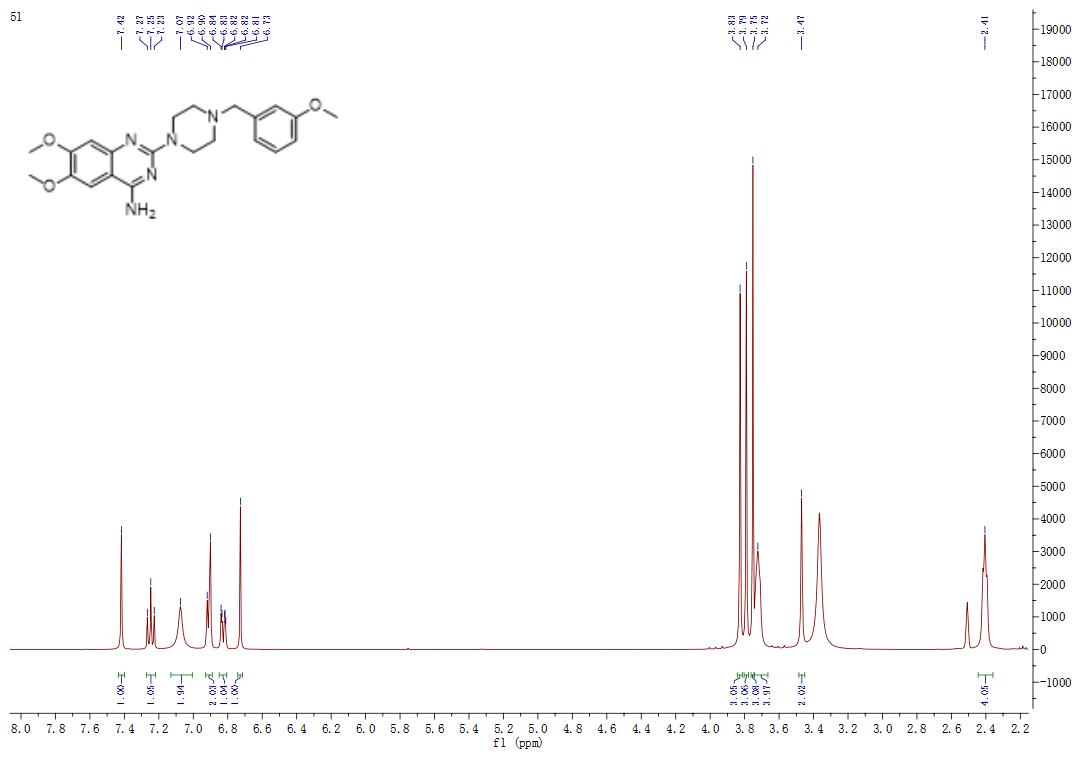
**

**
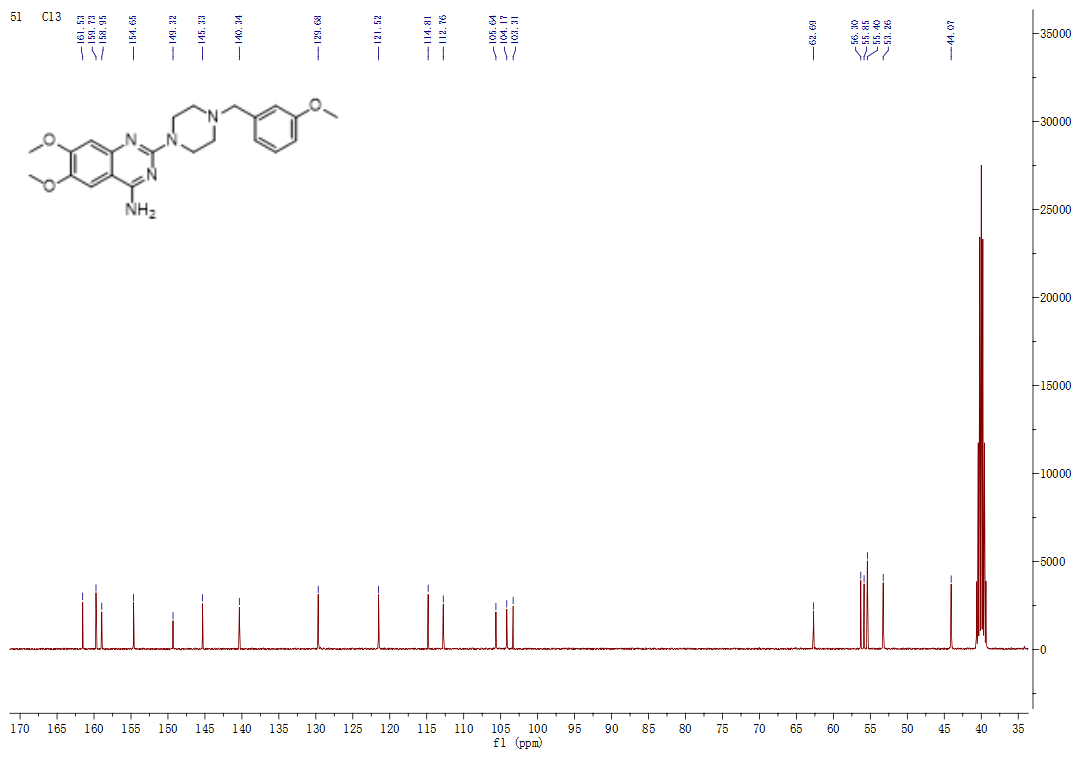
**

**Figure S12.** NMR spectrum of compound (**5l**)

**
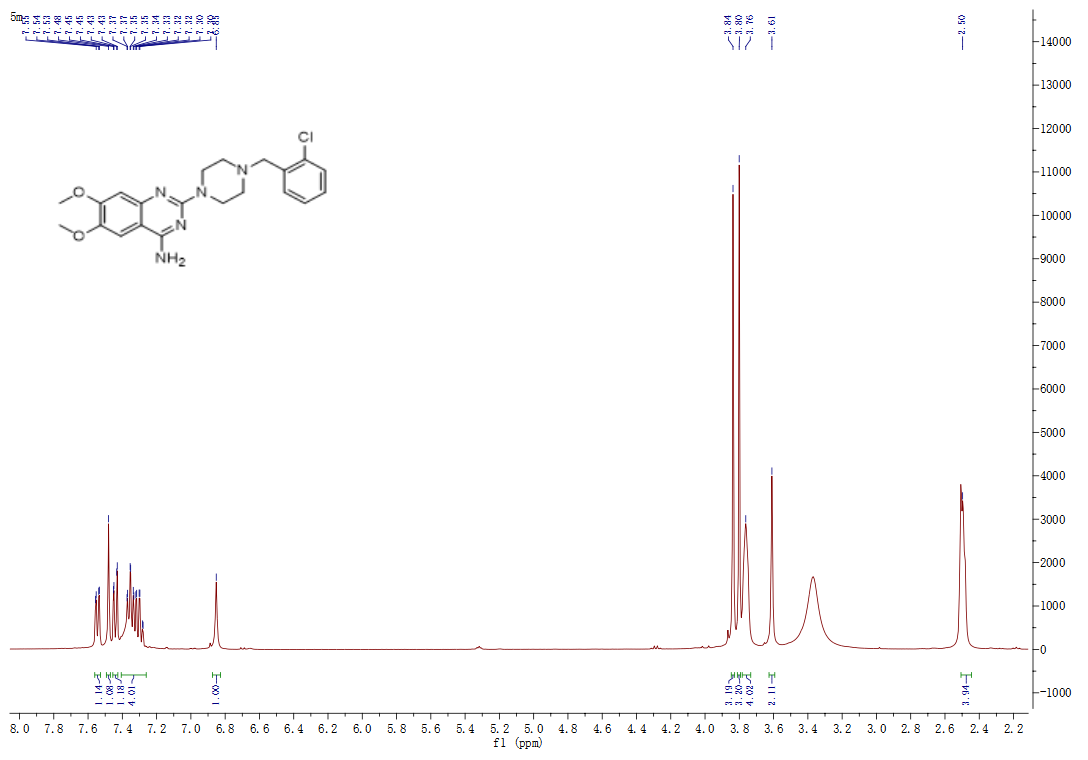
**

**
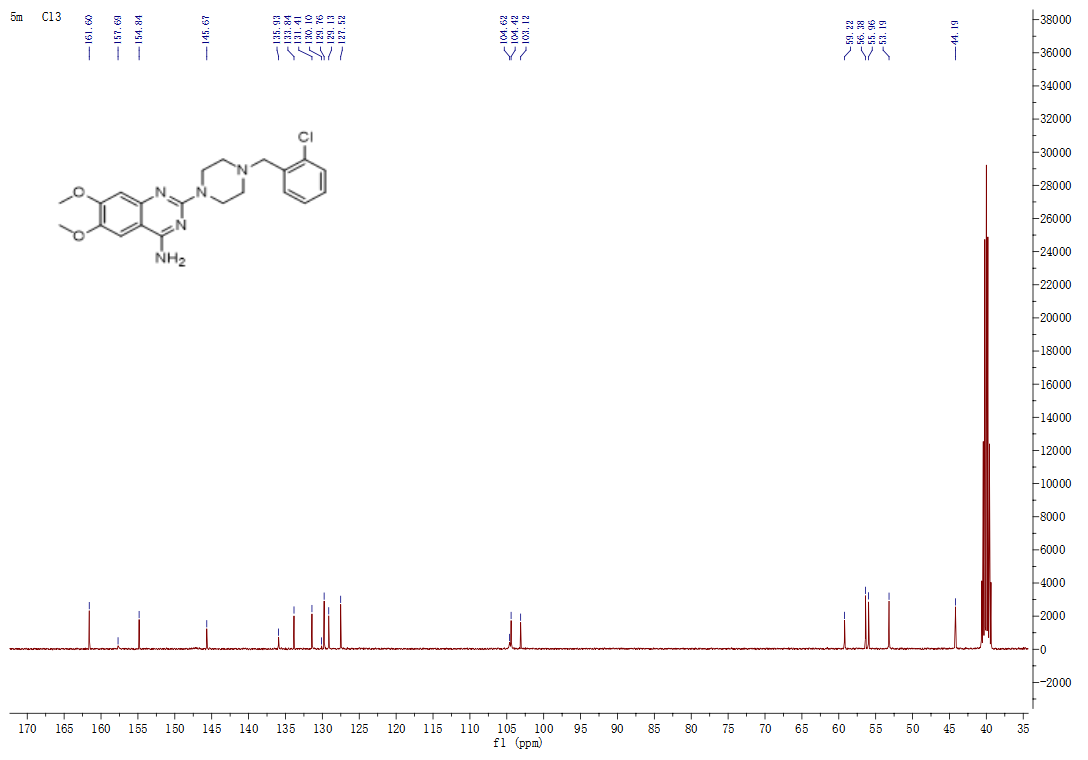
**

**Figure S13.** NMR spectrum of compound (**5m**)

**
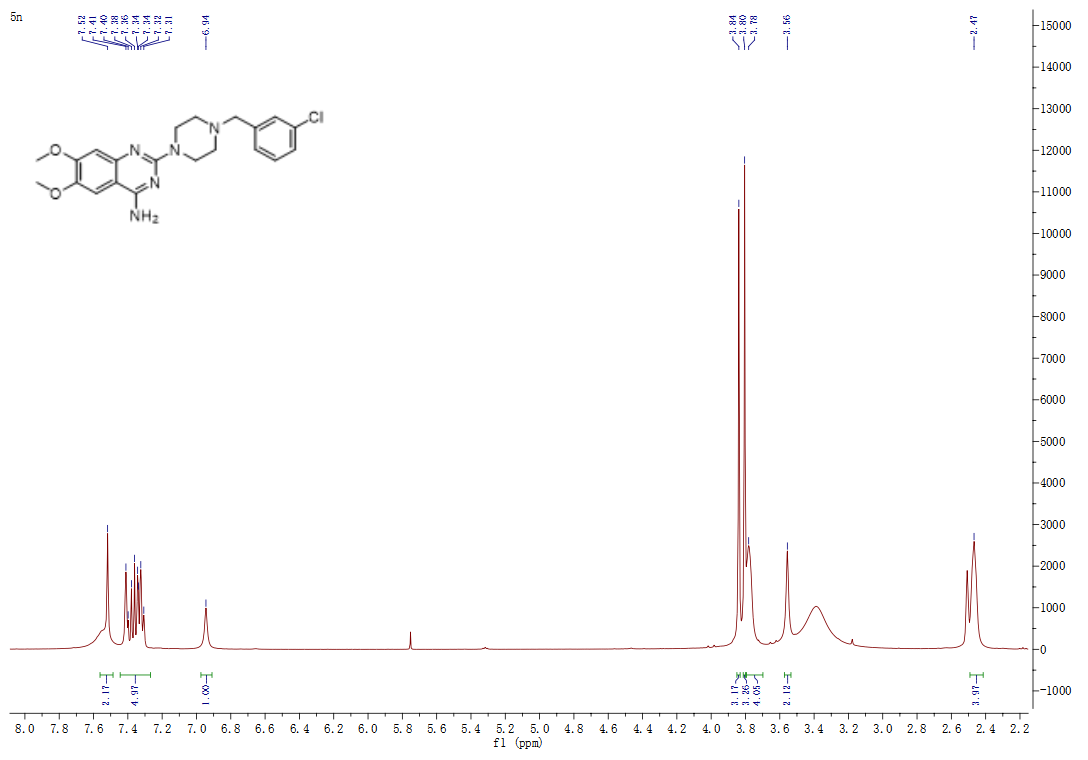
**

**
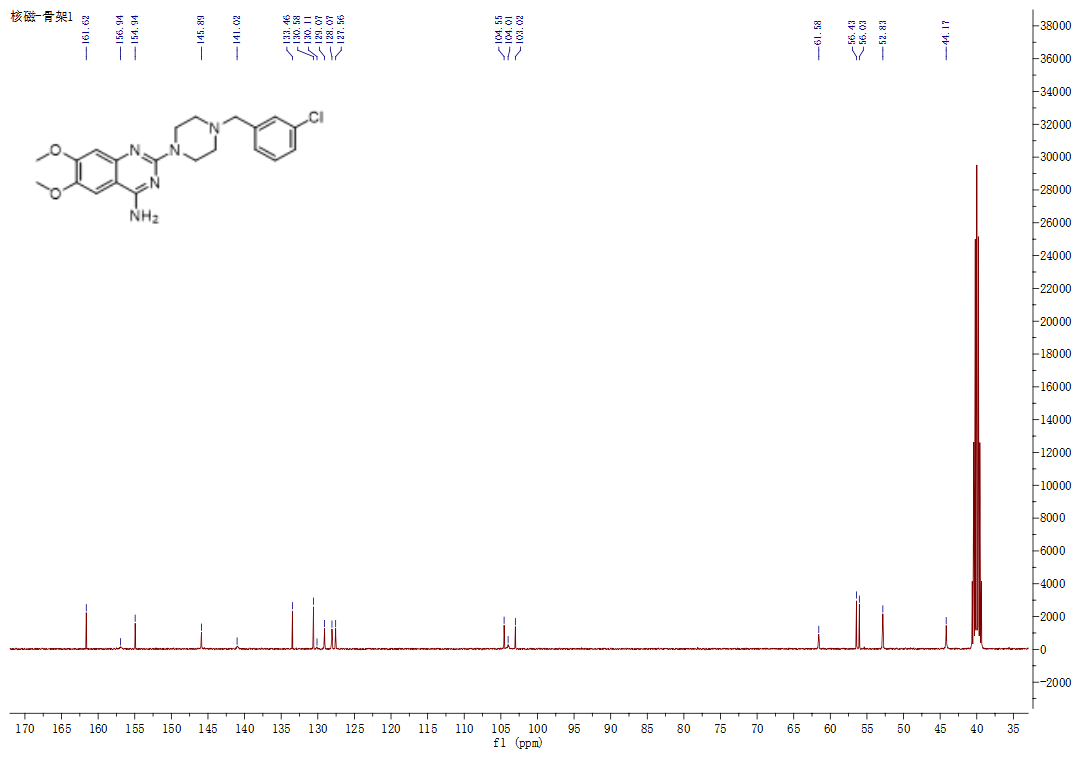
**

**Figure S14.** NMR spectrum of compound (**5n**)

**
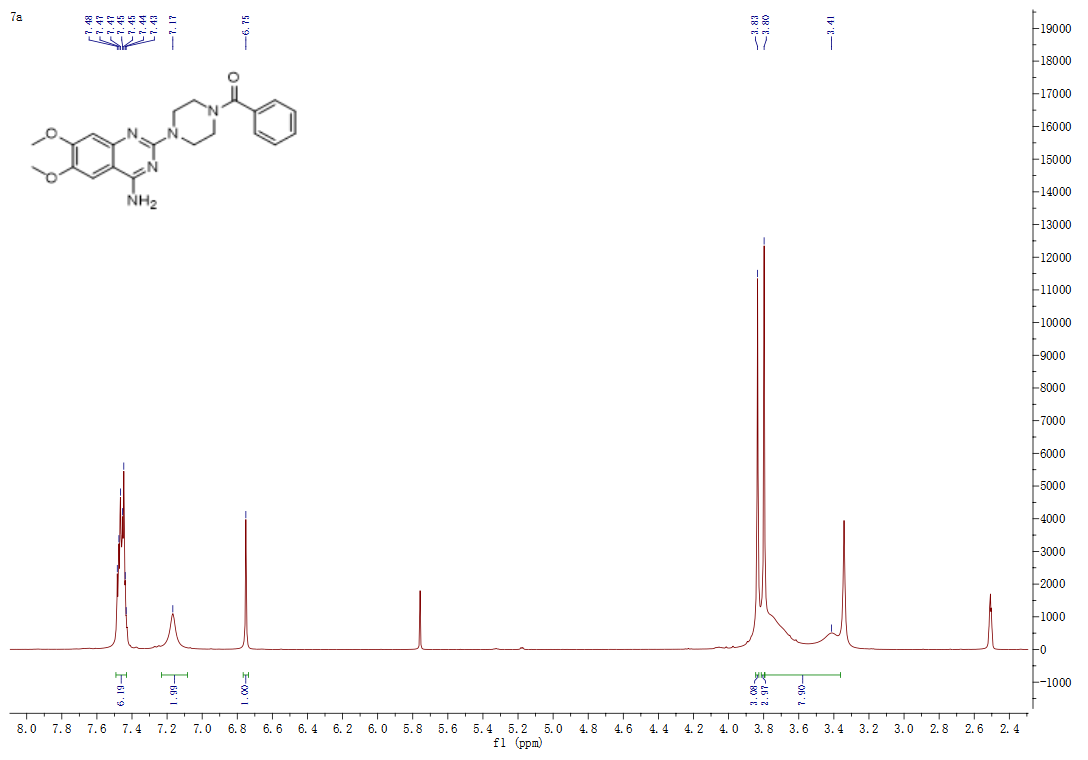
**

**
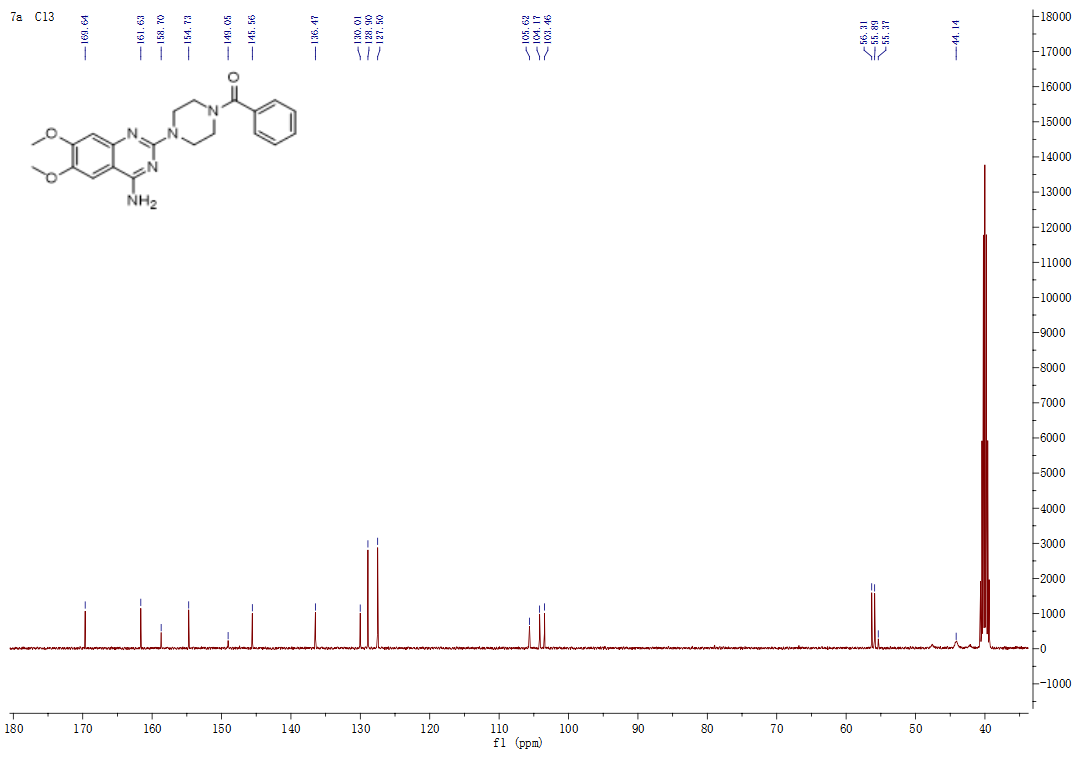
**

**Figure S15.** NMR spectrum of compound (**7a**)

**
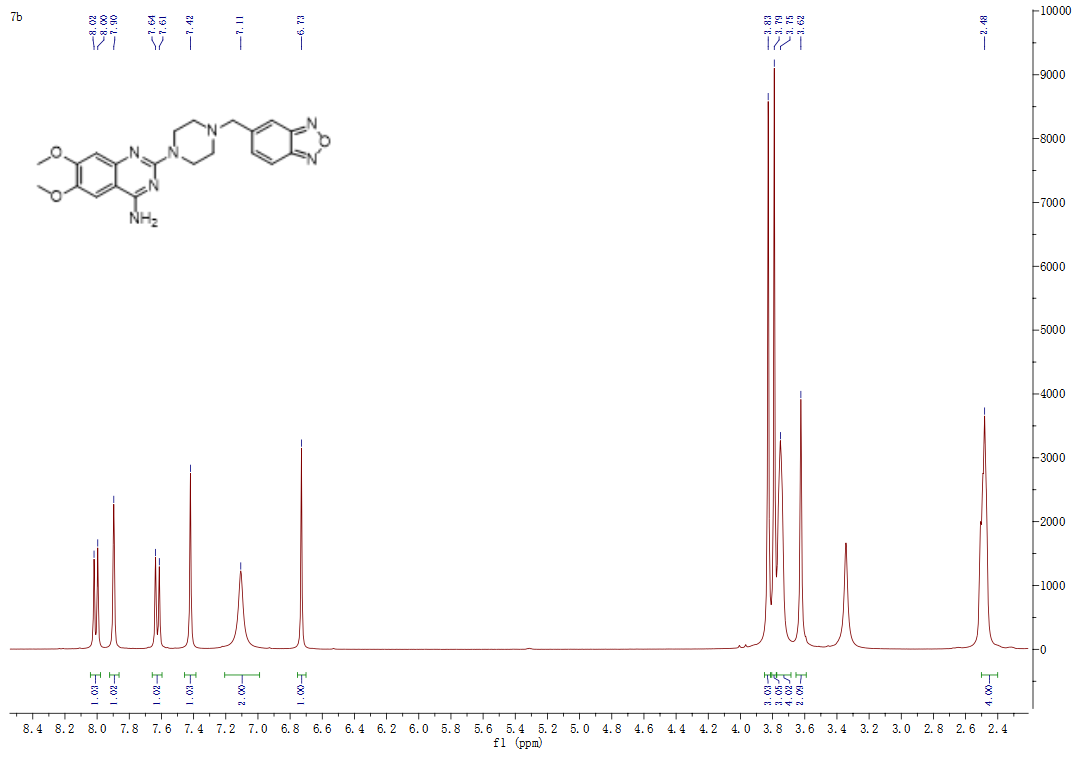
**

**
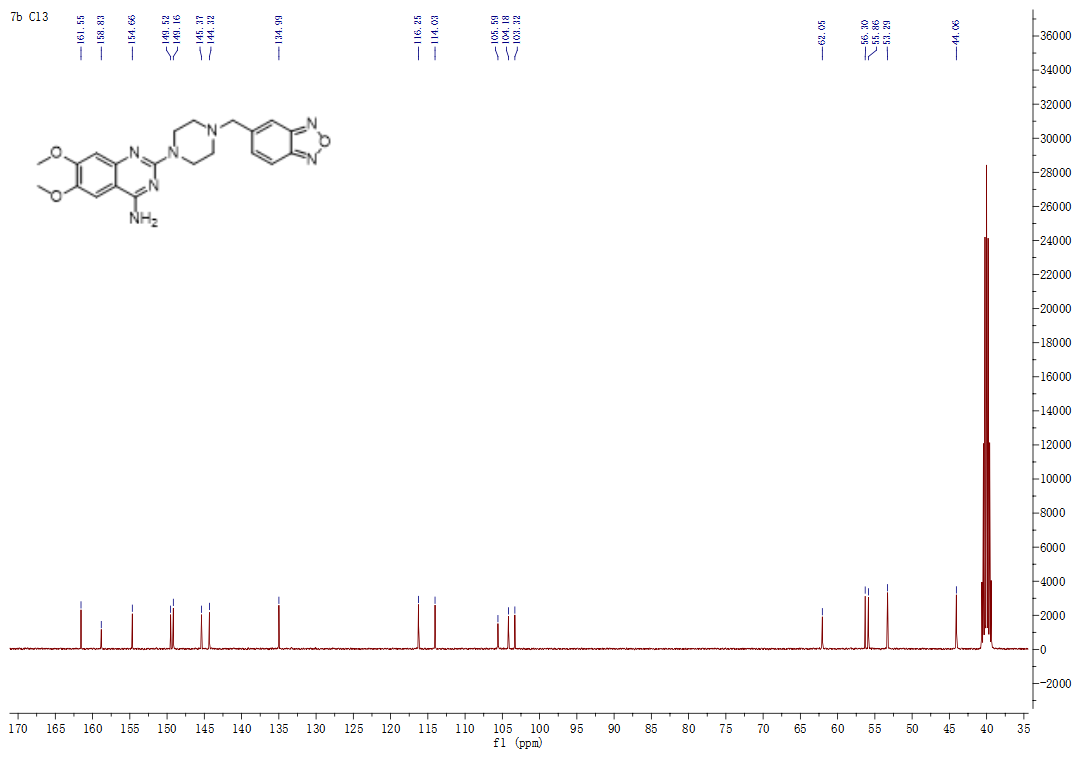
**

**Figure S16.** NMR spectrum of compound (**7b**)

**
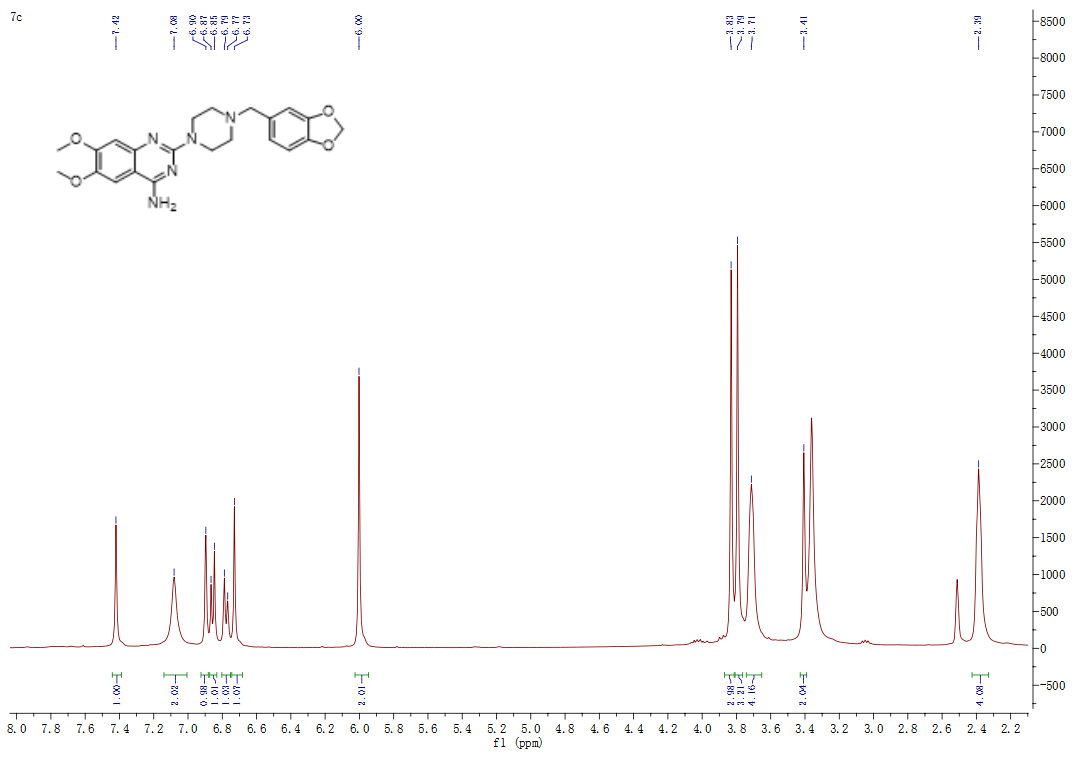
**

**
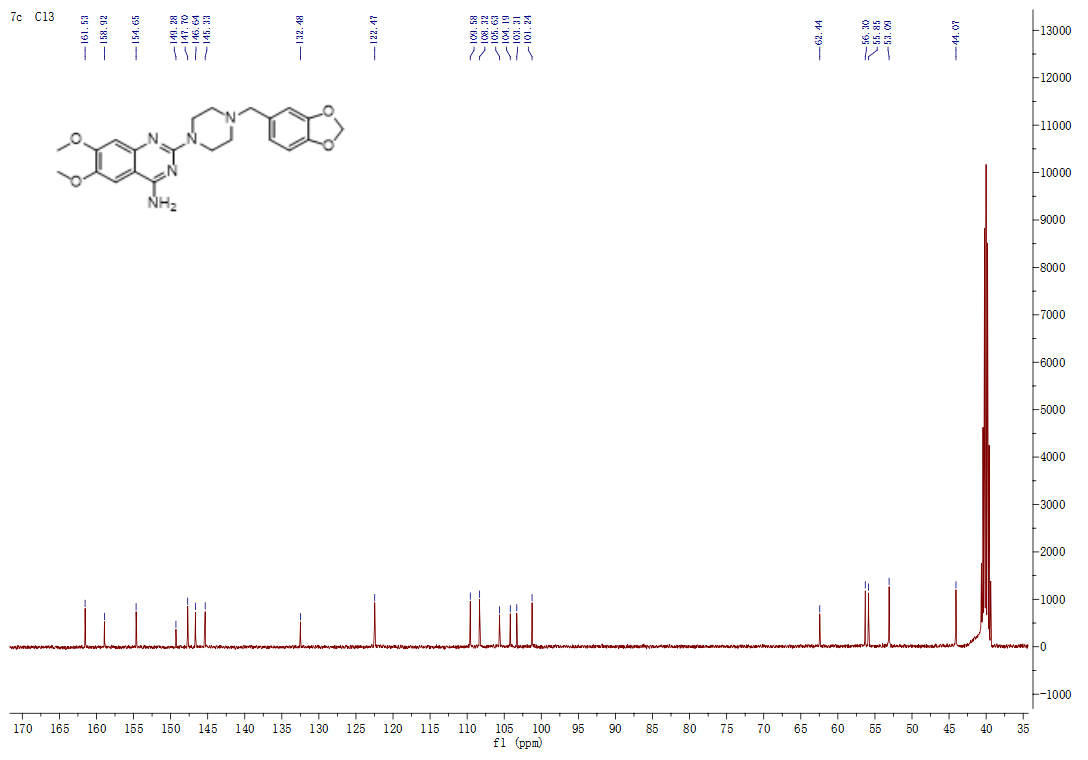
**

**Figure S17.** NMR spectrum of compound (**7c**)


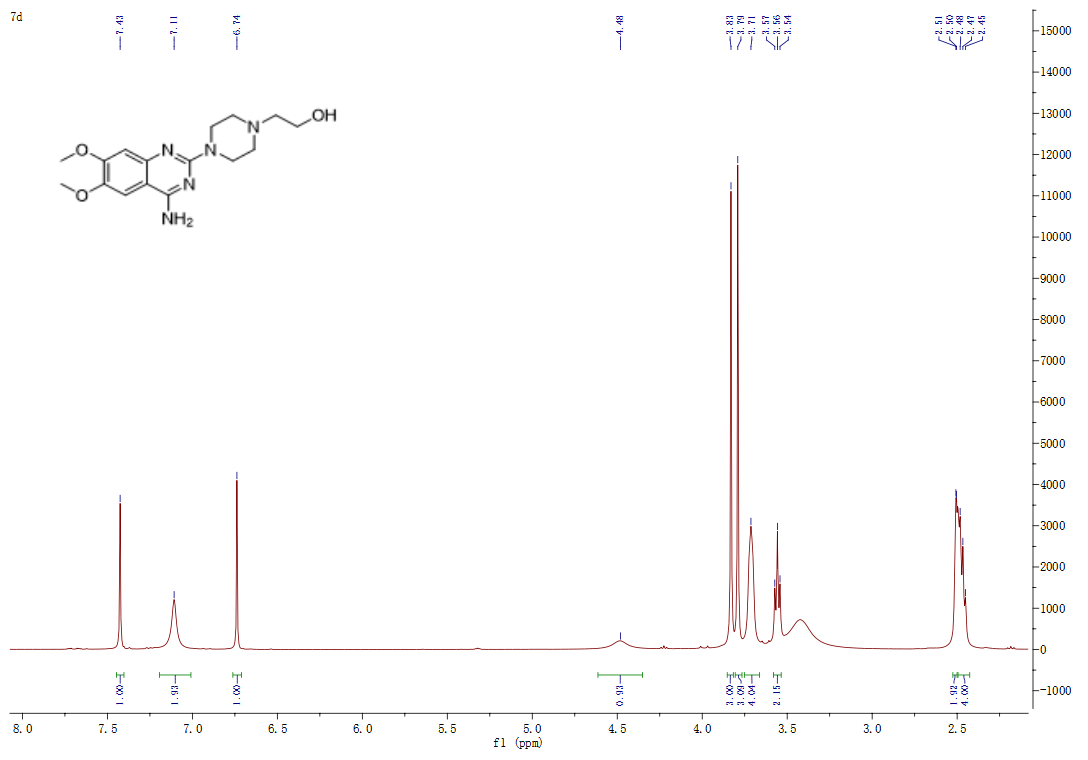


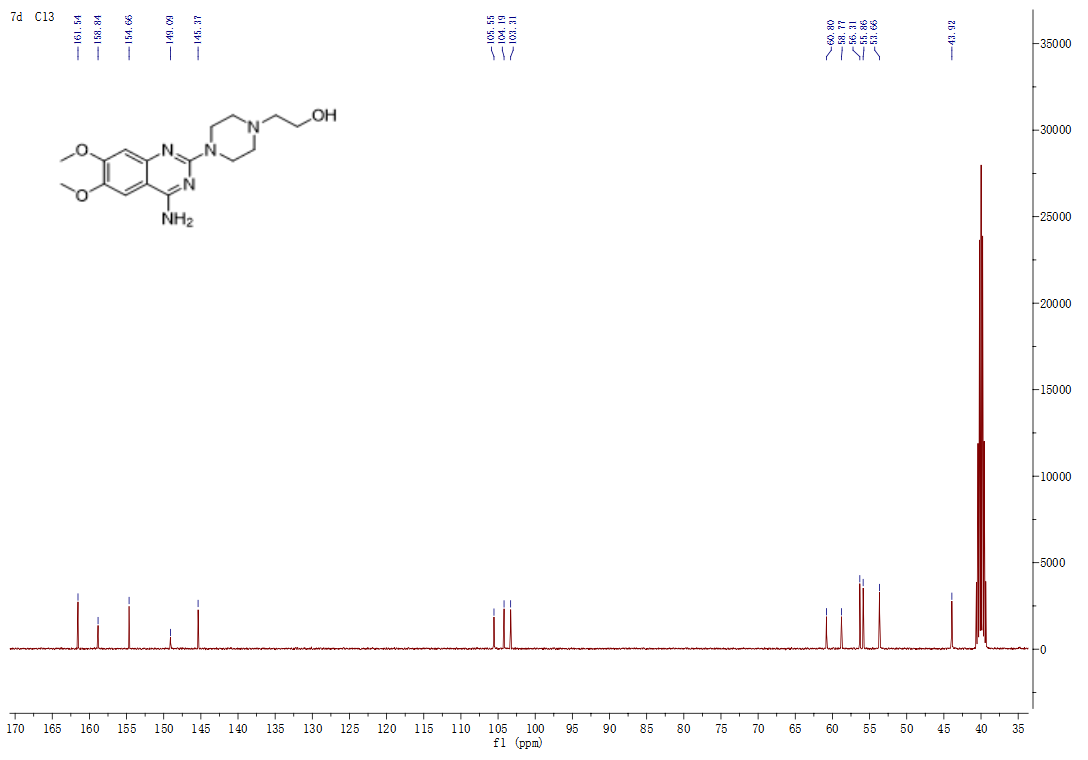


**Figure S18.** NMR spectrum of compound (**7d**)

**
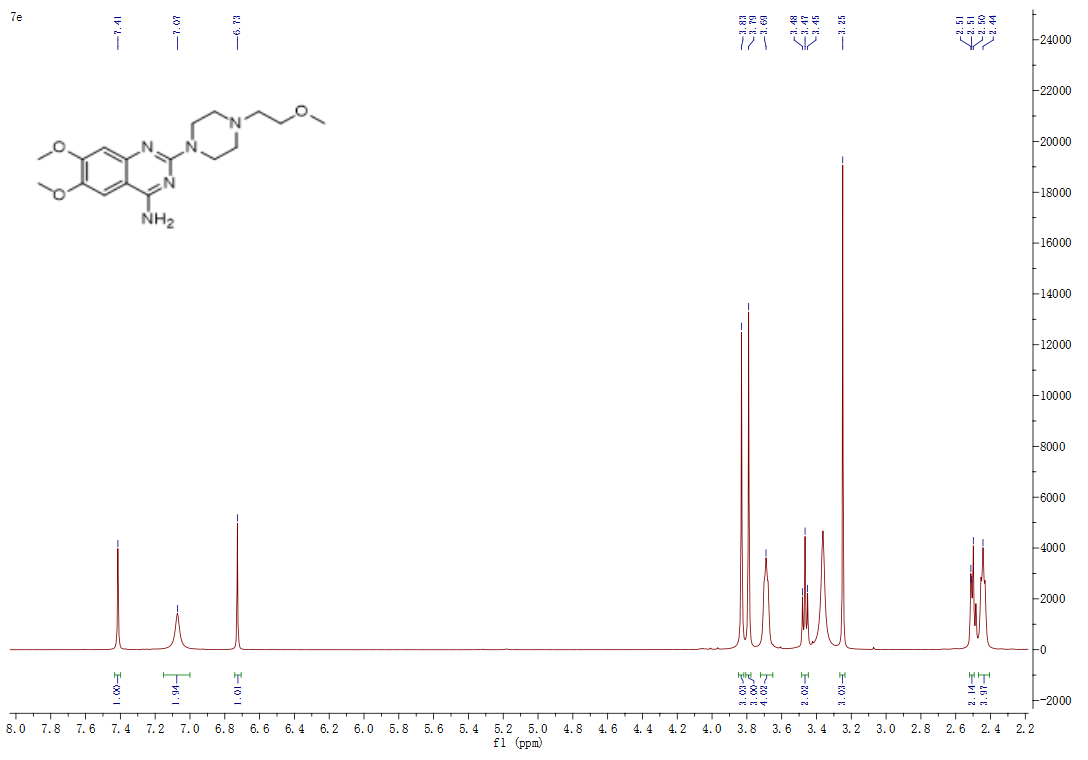
**

**
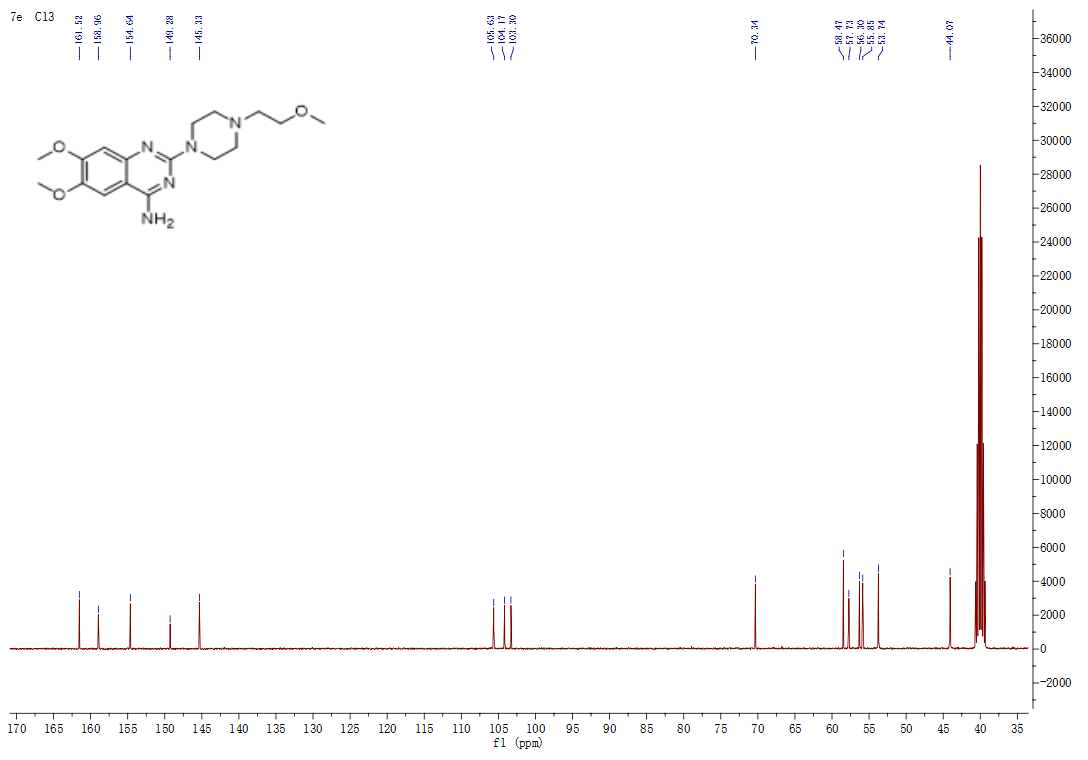
**

**Figure S19.** NMR spectrum of compound (**7e**)

**
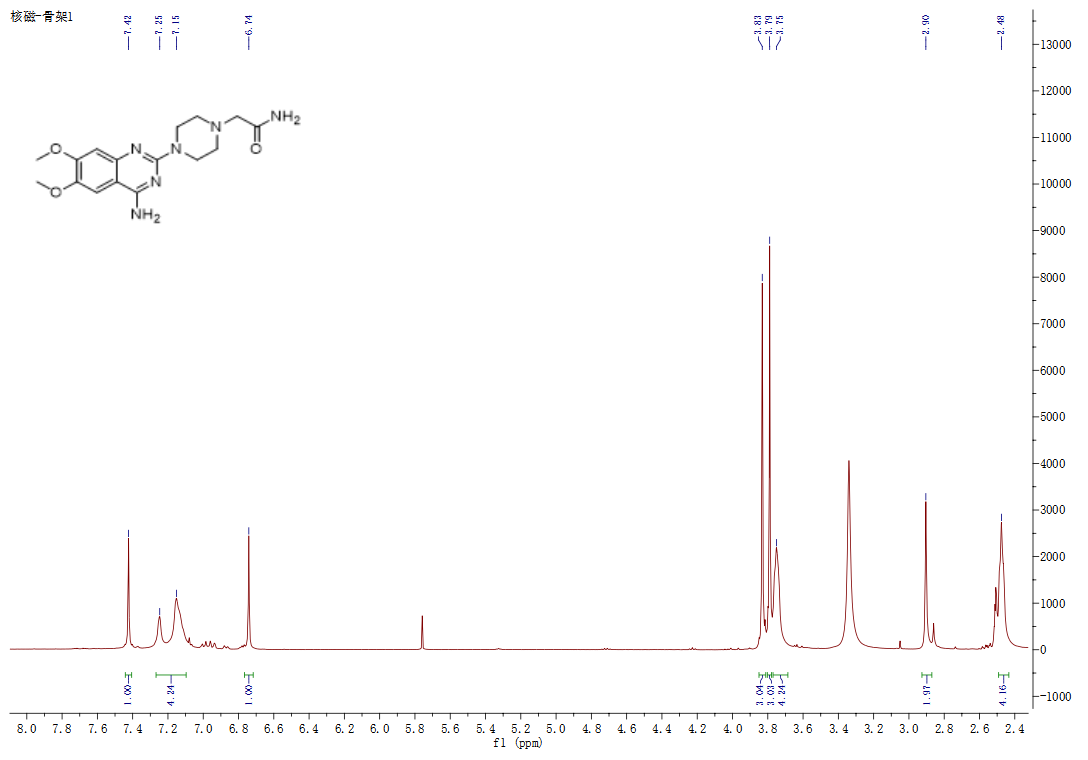
**

**
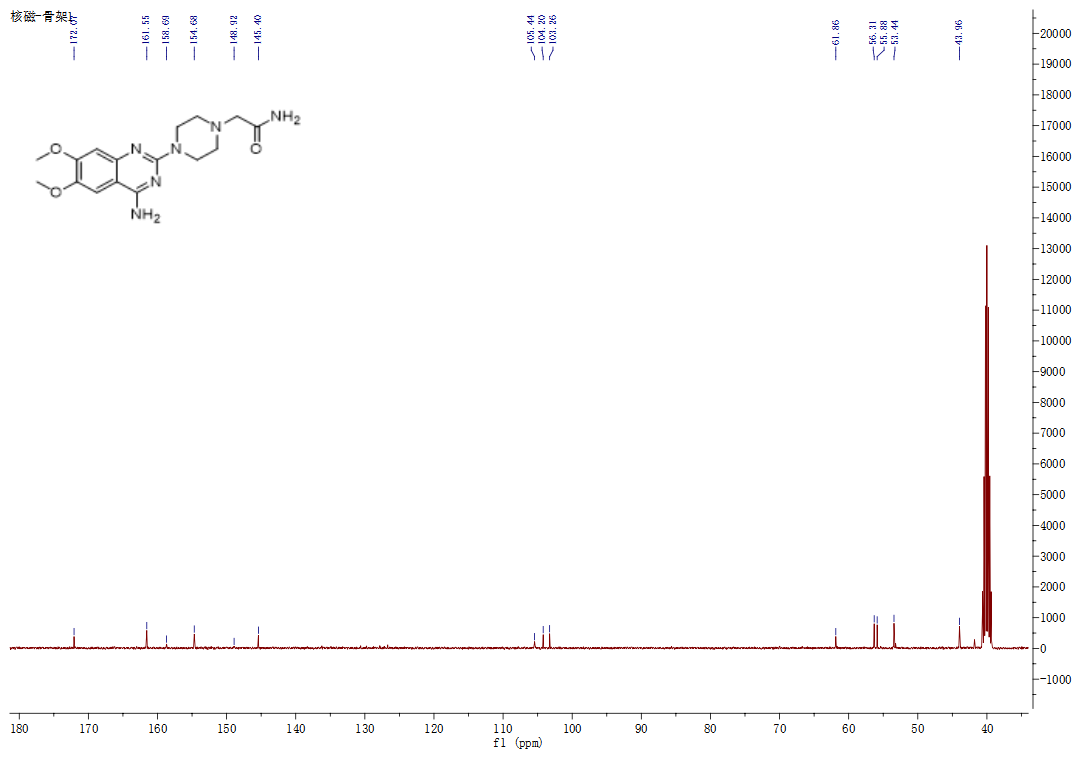
**

**Figure S20.** NMR spectrum of compound (**7f**)

**
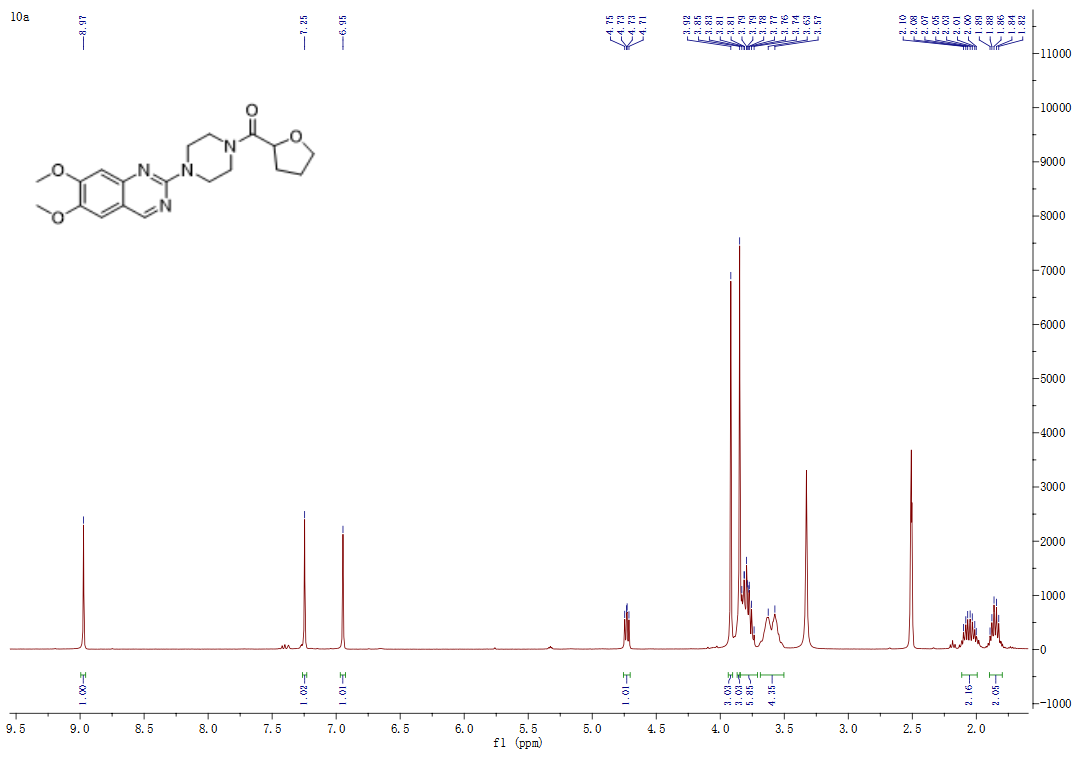
**

**
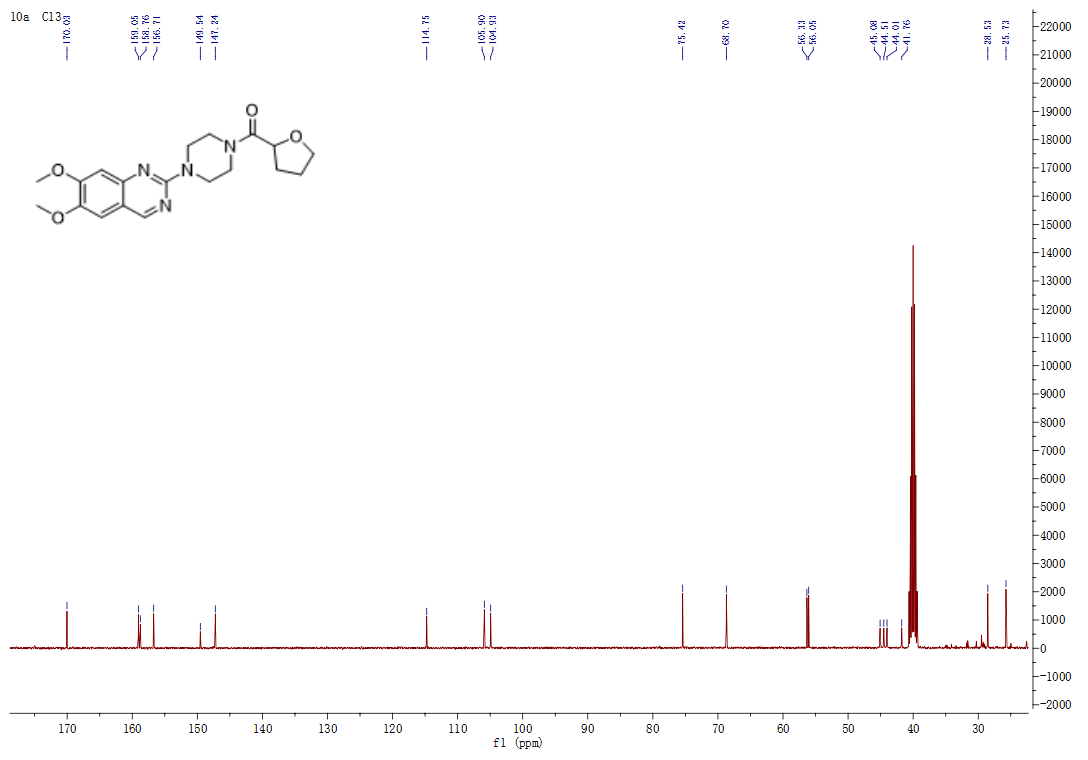
**

**Figure S21.** NMR spectrum of compound (**10a**)

**
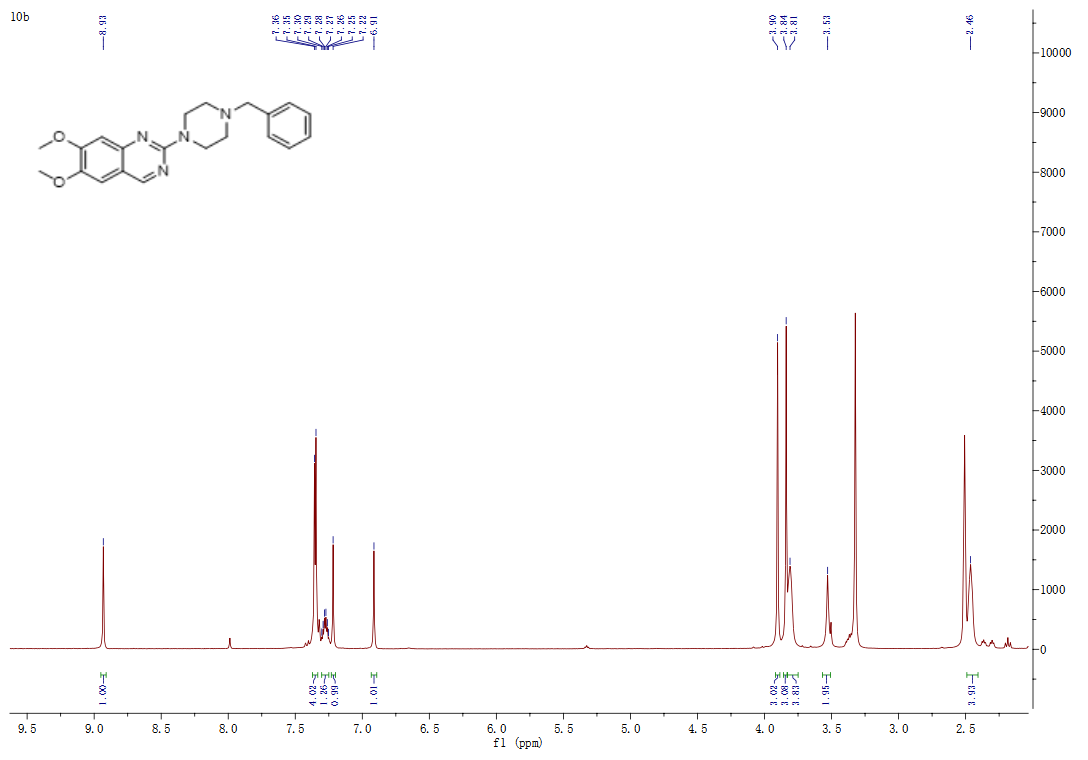
**

**
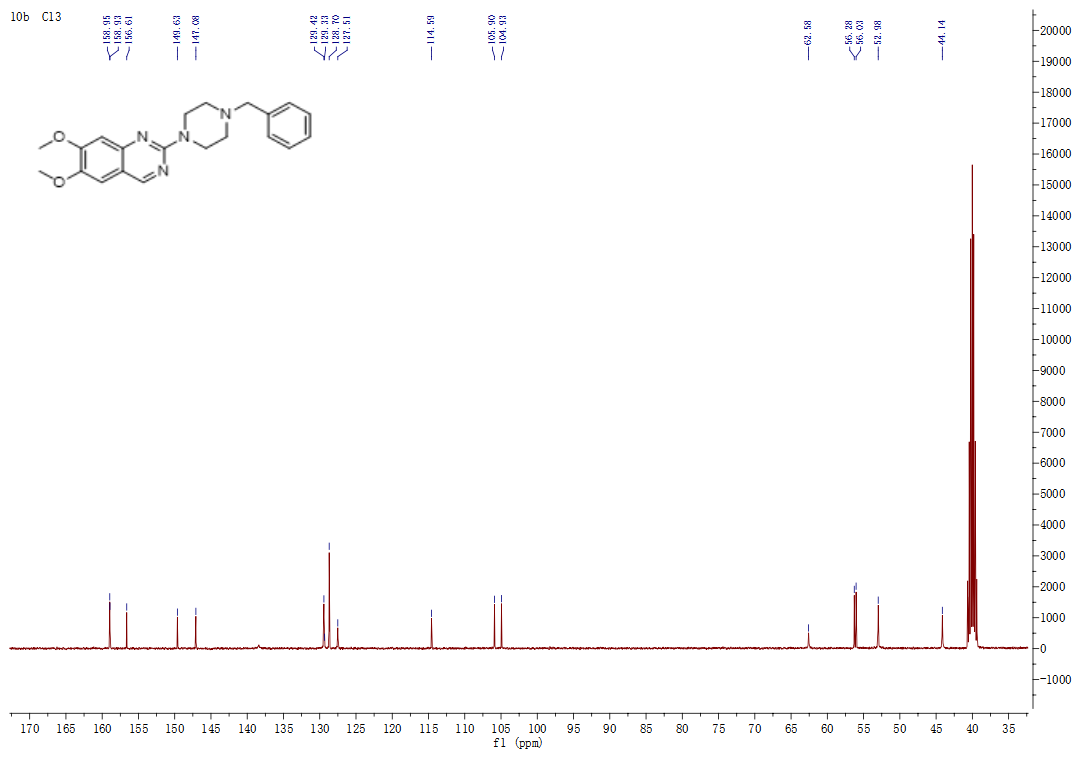
**

**Figure S22.** NMR spectrum of compound (**10b**)

**
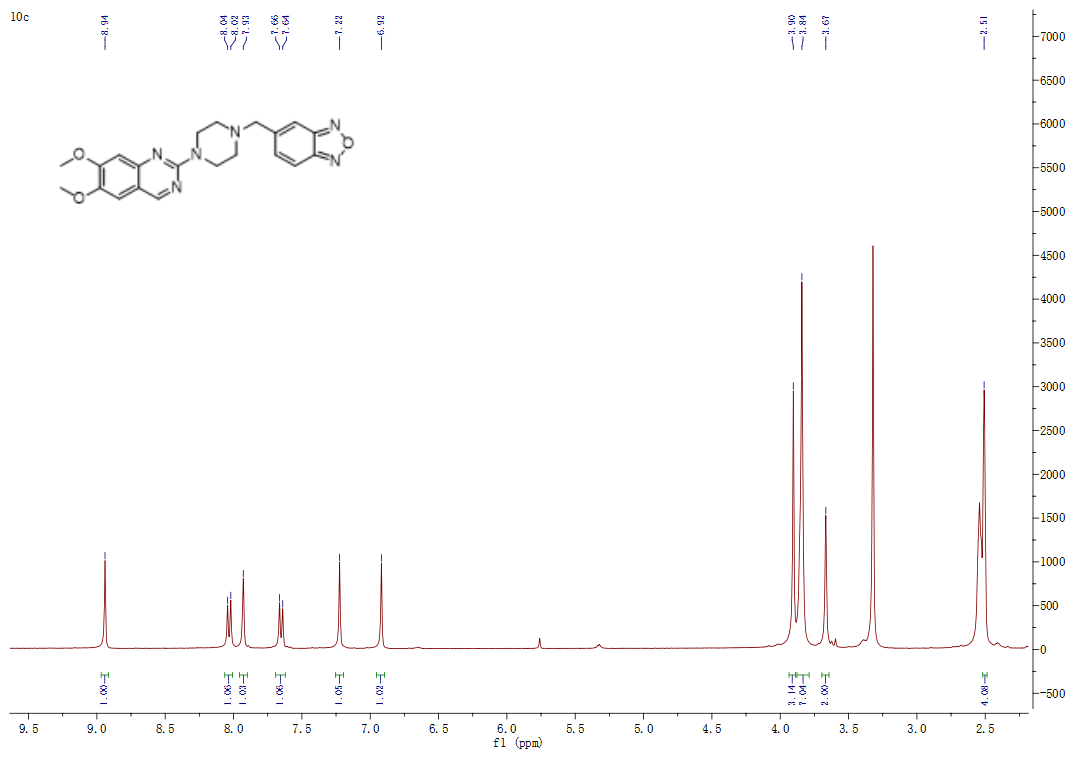
**

**
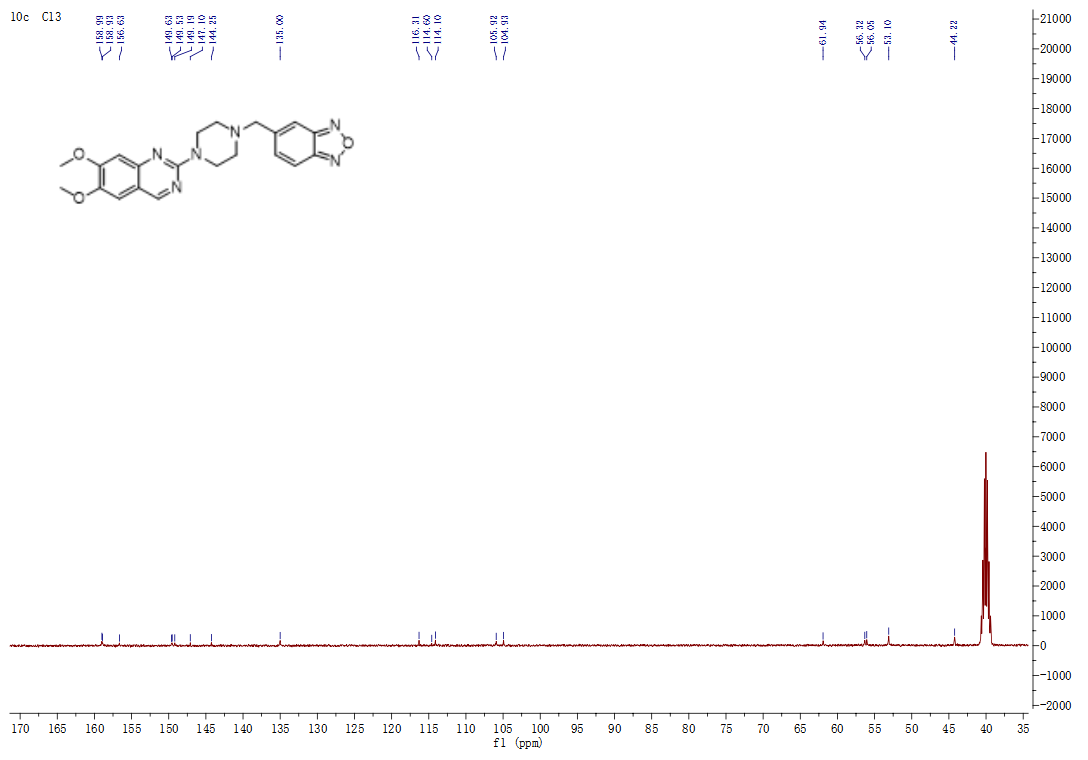
**

**Figure S23.** NMR spectrum of compound (**10c**)

**
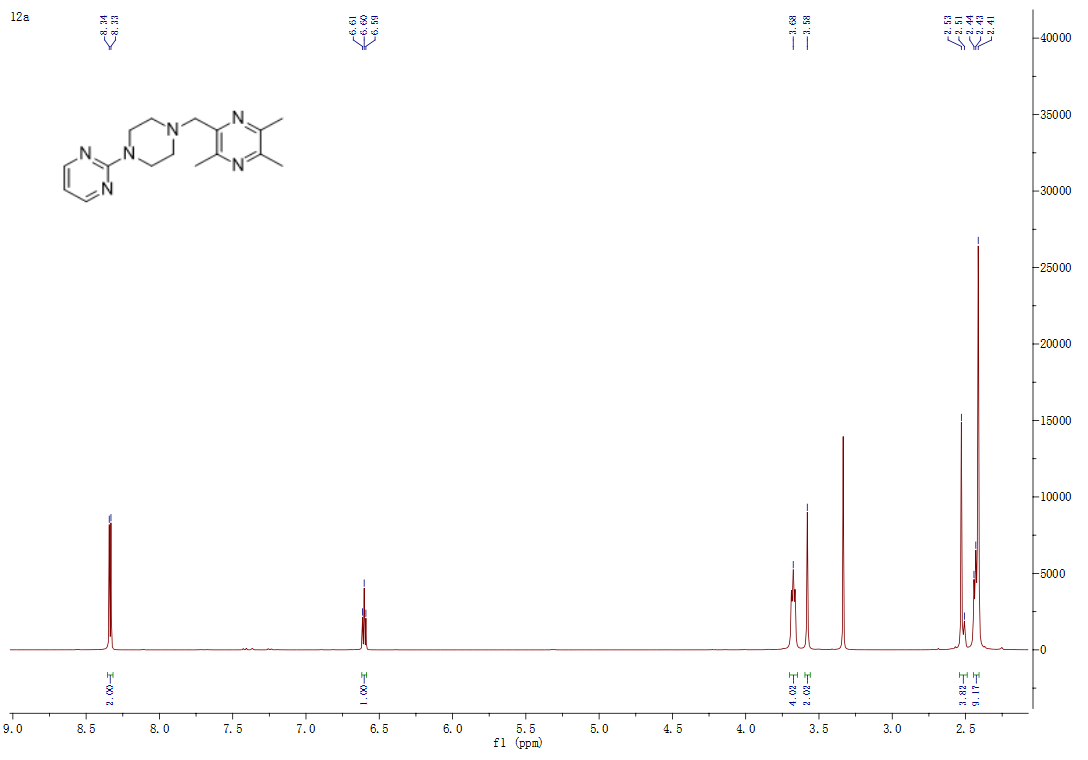
**

**
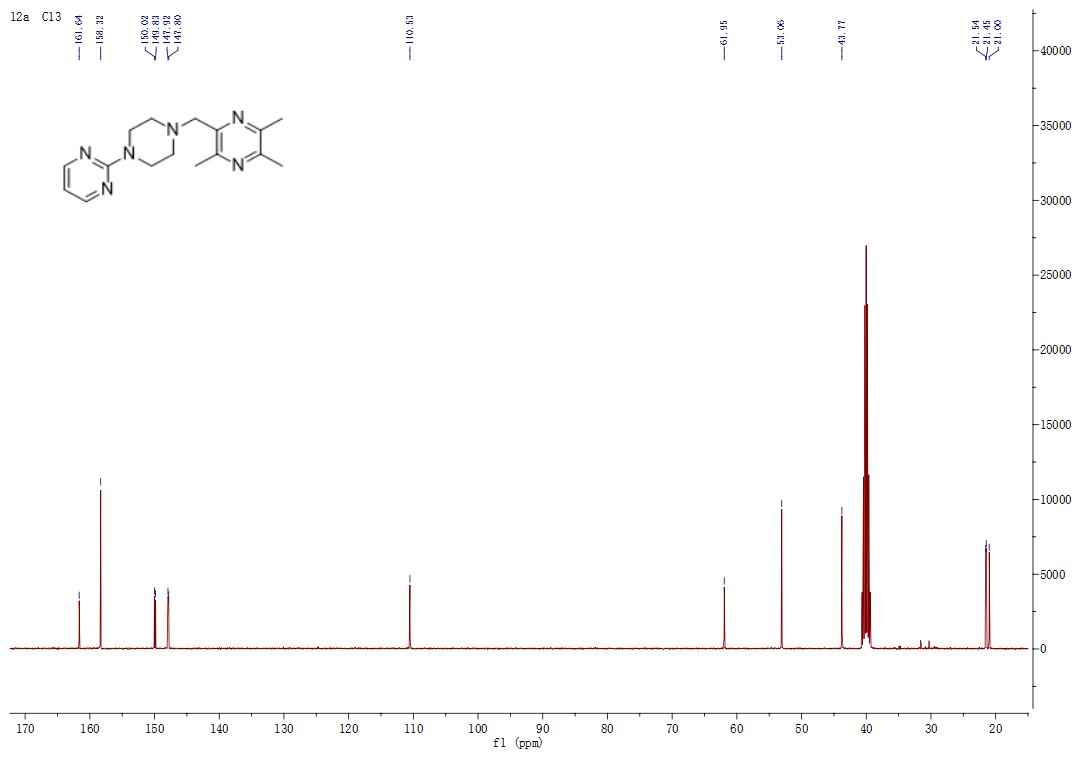
**

**Figure S24.** NMR spectrum of compound (**12a**)

**
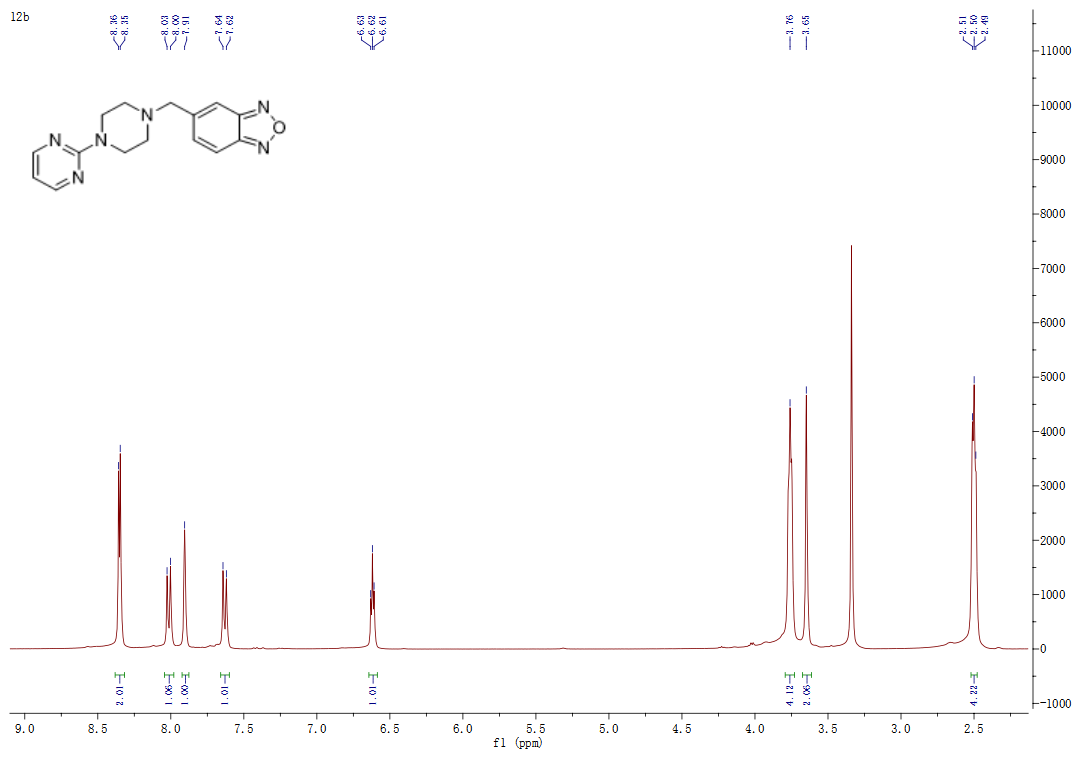
**

**
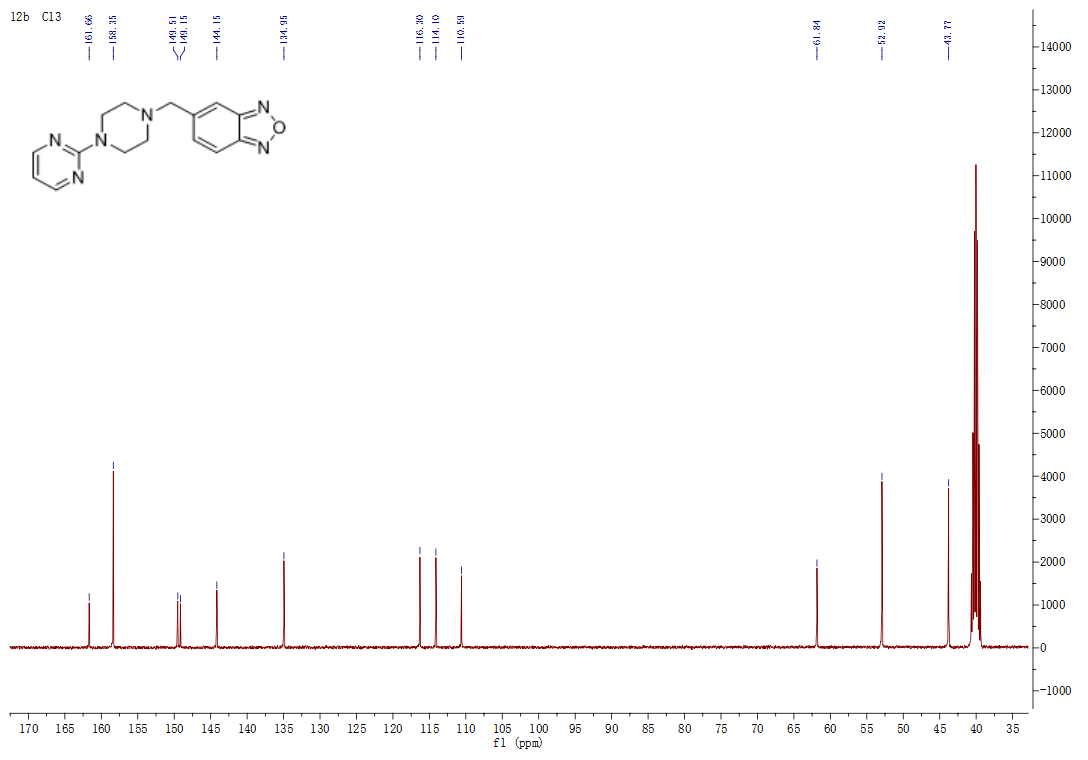
**

**Figure S25.** NMR spectrum of compound (**12b**)

# Figures


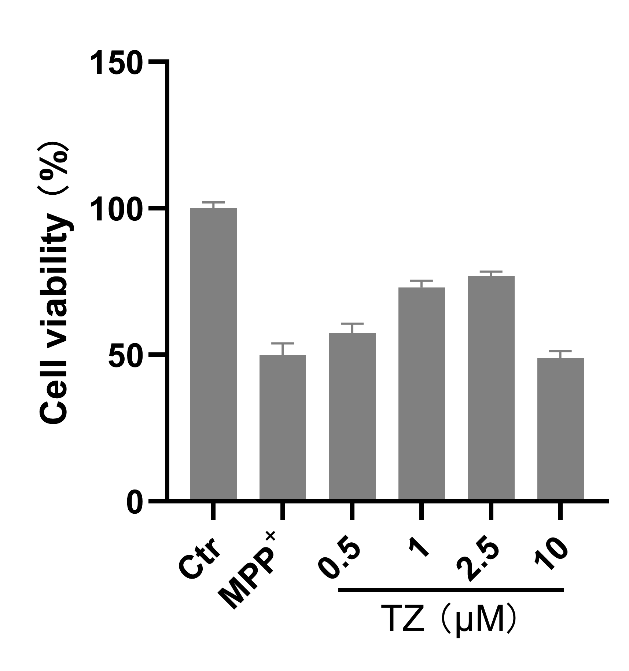


**Figure S26.** Different concentrations of terazosin on the viability of SH-SY5Y cells induced by MPP^+^.

# Tables

**Table S1**. Neuroprotective effects of target compounds on SH-SY5Y cells differentiated by RA/TPA.

| Compound | R_1_ | R_2_ | Survival rate (%) (2.5 μM) |
| --- | --- | --- | --- |
| Ctr | - | - | 100.00±4.84 |
| MPP^+^ | - | - | 54.27±3.27 |
| terazosin |  | NH_2_ | 75.06±1.84 |
| **5a** |  | NH_2_ | 57.52±2.29 |
| **5l** |  | NH_2_ | 67.78±0.62 |
| **7d** |  | NH_2_ | 72.12±1.13 |
| **10a** |  | H | 76.46±8.74 |
| **12a** |  | | 73.23±3.18 |
| **12b** |  | | 77.20±1.90 |

After being cultured in 96-well plates (1000 cells per well), the cells were first induced with 10 μM RA for 3 days, followed by 80 nM TPA for 3 days. After cell differentiation, compounds were incubated in the same manner as in Section 2.4. Then add 1 mM MPP^+^ and continue to incubate for 48 hours. After incubation, the same method as in Section 2.4 was used to detect cell viability.
